# Characterizing and Tailoring the Substrate Profile of a γ-Glutamyltransferase Variant

**DOI:** 10.1021/acssynbio.4c00364

**Published:** 2024-08-12

**Authors:** David Mueller, Remo Baettig, Tilmann Kuenzl, Emilio Rodríguez-Robles, Tania Michelle Roberts, Philippe Marlière, Sven Panke

**Affiliations:** † Department of Biosystems Science and Engineering, ETH Zürich, 4056 Basel, Switzerland; ‡ TESSSI, The European Syndicate of Synthetic Scientists and Industrialists, 75002 Paris, France

**Keywords:** xenobiology, xenomolecules, synthetic transport system, synthetic biology, directed evolution, enzymatic release

## Abstract

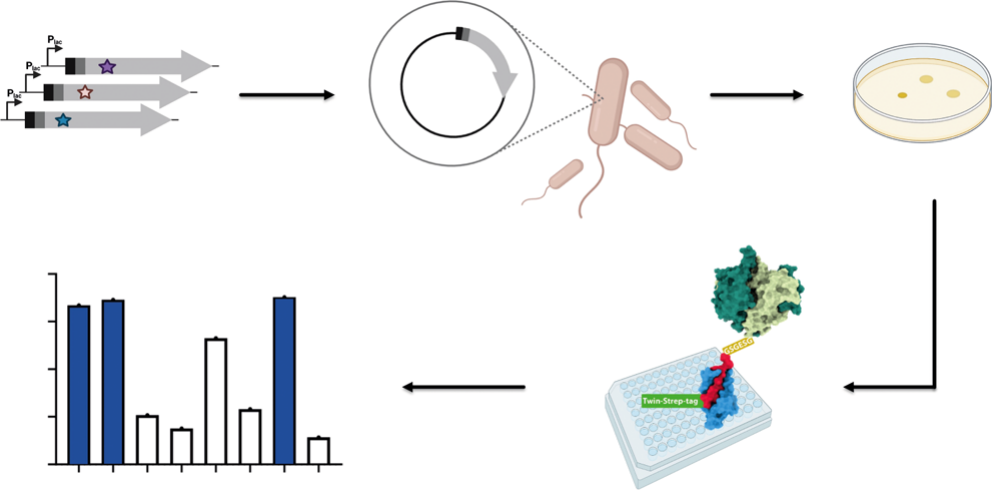

Xenobiology is an emerging field that focuses on the extension and redesign of biological systems through the use of laboratory-derived xenomolecules, which are molecules that are new to the metabolism of the cell. Despite the enormous potential of using xenomolecules in living organisms, most noncanonical building blocks still need to be supplied externally, and often poor uptake into cells limits wider applicability. To improve the cytosolic availability of noncanonical molecules, a synthetic transport system based on portage transport was developed, in which molecules of interest “cargo” are linked to a synthetic transport vector that enables piggyback transport through the alkylsulfonate transporter (SsuABC) of *Escherichia coli*. Upon cytosolic delivery, the vector-cargo conjugate is enzymatically cleaved by GGT_xe_, leading to the release of the cargo molecule. To deepen our understanding of the synthetic transport system, we focused on the characterization and further development of the enzymatic cargo release step. Hence, the substrate scope of GGT_xe_ was characterized using a library of structurally diverse vector-cargo conjugates and MS/MS-based quantification of hydrolysis products in a kinetic manner. The resulting substrate tolerance characterization revealed that vector-amino acid conjugates were significantly unfavored. To overcome this shortcoming, a selection system based on metabolic auxotrophy complementation and directed evolution of GGT_xe_ was established. In a directed evolution campaign, we improved the enzymatic activity of GGT_xe_ for vector-amino acid conjugates and revealed the importance of residue D386 in the cargo unloading step.

## Introduction

In the field of synthetic biology, the repertoire of natural biological building blocks is actively expanded by their noncanonical counterparts, which permits access to novel functional groups, increased bioorthogonality, and extended diversity. Using these lab-derived xenomolecules, which are molecules new to the metabolism of the cell, has led to the discovery of new metabolic pathways,^1^ novel genetic codes,^2−5^ and new-to-nature enzymatic catalysts.^6^ However, the incorporation of xenomolecules into proteins and metabolic pathways can be challenging due to limited membrane permeability.^7^ The lack of suitable transporters and undesirable molecular properties that prevent diffusion across the membranes are key factors when it comes to the cytosolic availability of xenomolecules in *Escherichia coli*.^8−11^

Different strategies to increase the cytosolic availability of noncanonical building blocks have been successfully applied, such as increased expression or engineering of transporters with relaxed substrate specificity.^12−14^ Other approaches directly circumvented the import problem by establishing a biosynthetic pathway for a nonproteogenic amino acid.^7^ Although those attempts were able to increase the bioavailability of xenomolecules, they were specific to only a single group of noncanonical molecules and could not serve as a more general solution to the problem.

**Figure 1:**
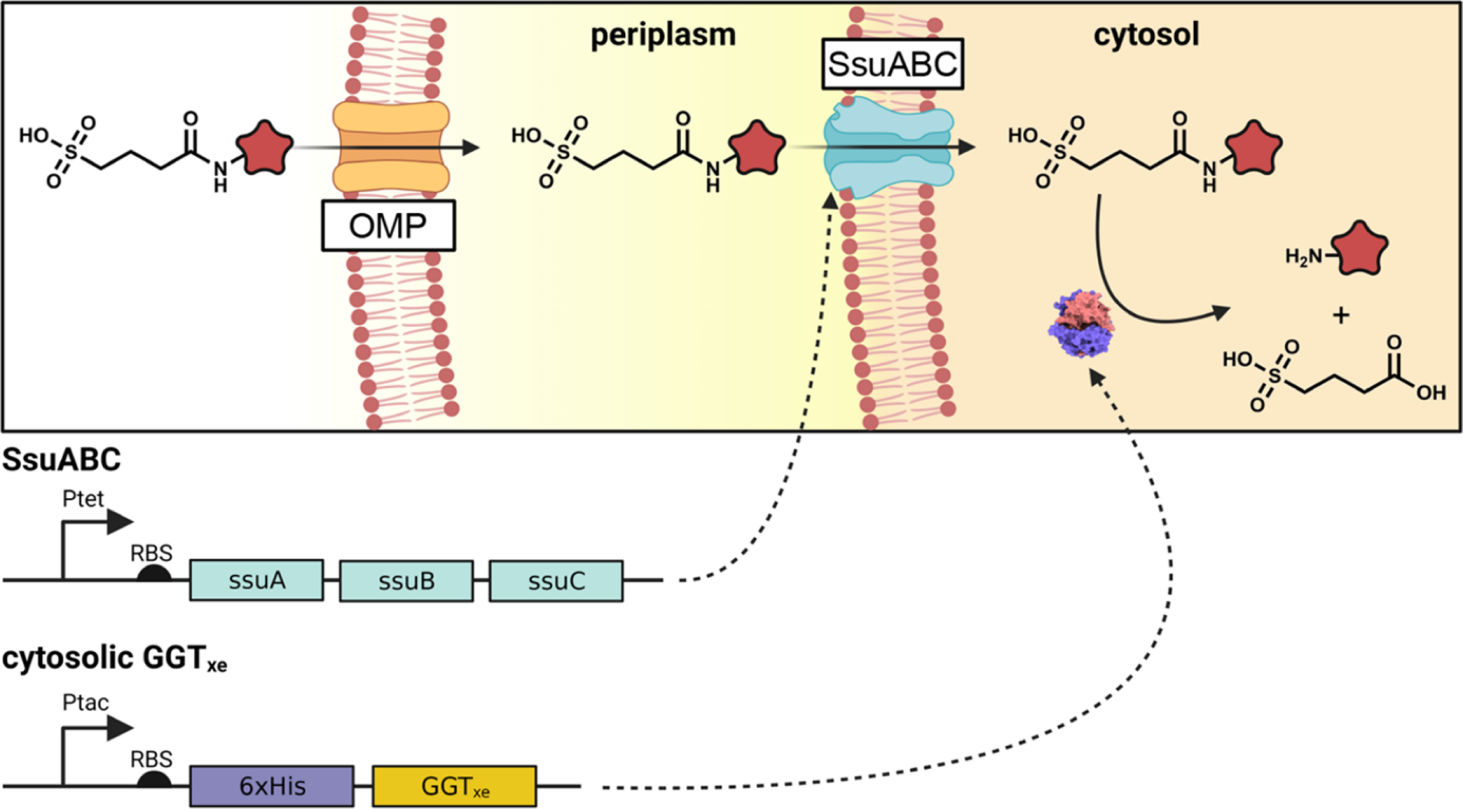
Summary of the sulfonate-based synthetic transport system for nonmembrane permeable molecules. Schematic illustration of the synthetic import system showing the uptake of a nonmembrane permeable molecule (cargo, red star) into the cytosol of *E. coli* (adapted from ref (15)). The cargo is bound to the vector 4-sulfobutanoic acid, passes the outer membrane through unspecific outer membrane pores (OMP), and is then imported by the alkylsulfonic acid transporter (SsuABC) with relaxed substrate specificity. In a second step, a cytosolic γ-glutamyltransferase variant derived from *Pseudomonas nitroreducens* (*Pn*GGT D406N, referred to as GGT_xe_) is used to hydrolyze the imported conjugate, thereby releasing the cargo.

In order to increase the cytosolic availability of xenomolecules in a more universal fashion, we developed a synthetic transport system that allowed for the specific uptake of xenomolecules into the cytosol of *E. coli*.^15^ The transport system follows a simple three-step procedure: (i) The amino-bearing molecule of interest “cargo” is chemically conjugated to the carboxyl group of 4-sulfobutanoic acid (SBA) “vector” via an amide bond. The vector-cargo conjugate is able to diffuse through the spacious outer membrane pores into the periplasm.^16,17^ (ii) Here, the SBA serves as a piggyback vector and enables the transport of the vector-cargo conjugate through the *E. coli* alkylsulfonate transporter (SsuABC). (iii) Upon cytosolic transport, an intracellularly relocated variant of the γ-glutamyltransferase of *Pseudomonas nitroreducens* (PnGGT D406N, referred to as GGT_xe_) is used to hydrolyze the amide bond. This leads to the release of the cargo molecule (Figure 1) and, thereby, implements a system to specifically cleave a vector from a cargo molecule *in vivo*.

In most Gram-negative bacteria, γ-glutamyltransferases are located in the periplasmic space^18,19^ and maintain the enzymatic degradation of glutathione, thereby enabling the scavenging of released amino acids.^20^ Based on the highly conserved stacked αββα core fold and the usage of a N-terminal nucleophile during catalysis, GGTs are assigned to the N-terminal nucleophile hydrolase superfamily (Ntn-hydrolases).^21^ Catalysis leads to the formation of an acyl-intermediate and deacylation occurs by transferring the glutamyl-moiety to a suitable acceptor substrate or via hydrolysis by water.^22^ Most GGTs are synthesized as inactive precursors and undergo post-translational autocatalytic processing that results in the formation of two subunits, known as large and small subunit. Upon proper folding, a mature heterodimeric enzyme is formed,^23^ whose small subunit contains the catalytic nucleophile and 7 out of 8 substrate binding residues.^24,25^ Of particular interest is the GGT of *Pseudomonas nitroreducens* since it prefers hydrolysis over transferase activity and is known to exhibit broad substrate tolerance.^26^ Especially in the context of a synthetic transport system, high enzymatic activity for a broad variety of vector-cargo molecules is highly desirable. Although broad substrate tolerance was described for *Pn*GGT, only little is known about the substrates used by the GGT_xe_ variant. Since the hydrolysis activity of GGT_xe_ is essential for the proper functionality of the XenoImport system, a substrate tolerance characterization could help to reveal potential limitations and provide meaningful insights regarding the design of vector-cargo conjugates.

In this study, we therefore focused on the ability of GGT_xe_ to release a variety of cargoes *in vitro*. As such, we characterized the substrate promiscuity of GGT_xe_ and observed reduced activity for most conjugates that consisted of an amino acid as a cargo molecule. In order to improve the hydrolysis activity of GGT_xe_ for amino acid-containing conjugates, a directed evolution campaign based on metabolic auxotrophy complementation was performed. In this campaign, a key residue (D386) involved in the hydrolysis of SBA- and glutaryl (GTA)-amino acid conjugates was identified. Furthermore, our findings demonstrate the broad substrate specificity of GGT_xe_, which makes it a very suitable cargo release enzyme for a synthetic transport system.

## Results

In order to characterize the substrate promiscuity of GGT_xe_, we compared initial hydrolysis rates of GGT_xe_ for members of a physicochemically diverse substrate library. Fundamentally, we are interested in the hydrolysis of conjugates containing the cargo moiety and the SBA vector. However, SBA-conjugates require custom synthesis; therefore, the screening of a substantial library is expensive. In contrast, GTA conjugates are easily accessible from various commercial sources, there is considerable similarity between these two molecular entities, and indeed it is known that a GGT mutant of *E. coli* (D433N, which corresponds to D406N in *Pn*GGT) hydrolyzes glutaryl-7-aminocephalosporanic acid.^27^ We purified GGTxe using an N-terminal Twin-streptag (Twin-streptag-SpyTag fused to GGTxe (tsGGTxe) (Figure S1) and previously showed that the hydrolysis activities between SBA- and GTA-conjugates are similar. Therefore, we acquired a substantial library of GTA-conjugates and combined them with some custom-synthesized compounds.

### Characterizing the Substrate Promiscuity of GGT_xe_ with GTA-Conjugates

**Figure 2:**
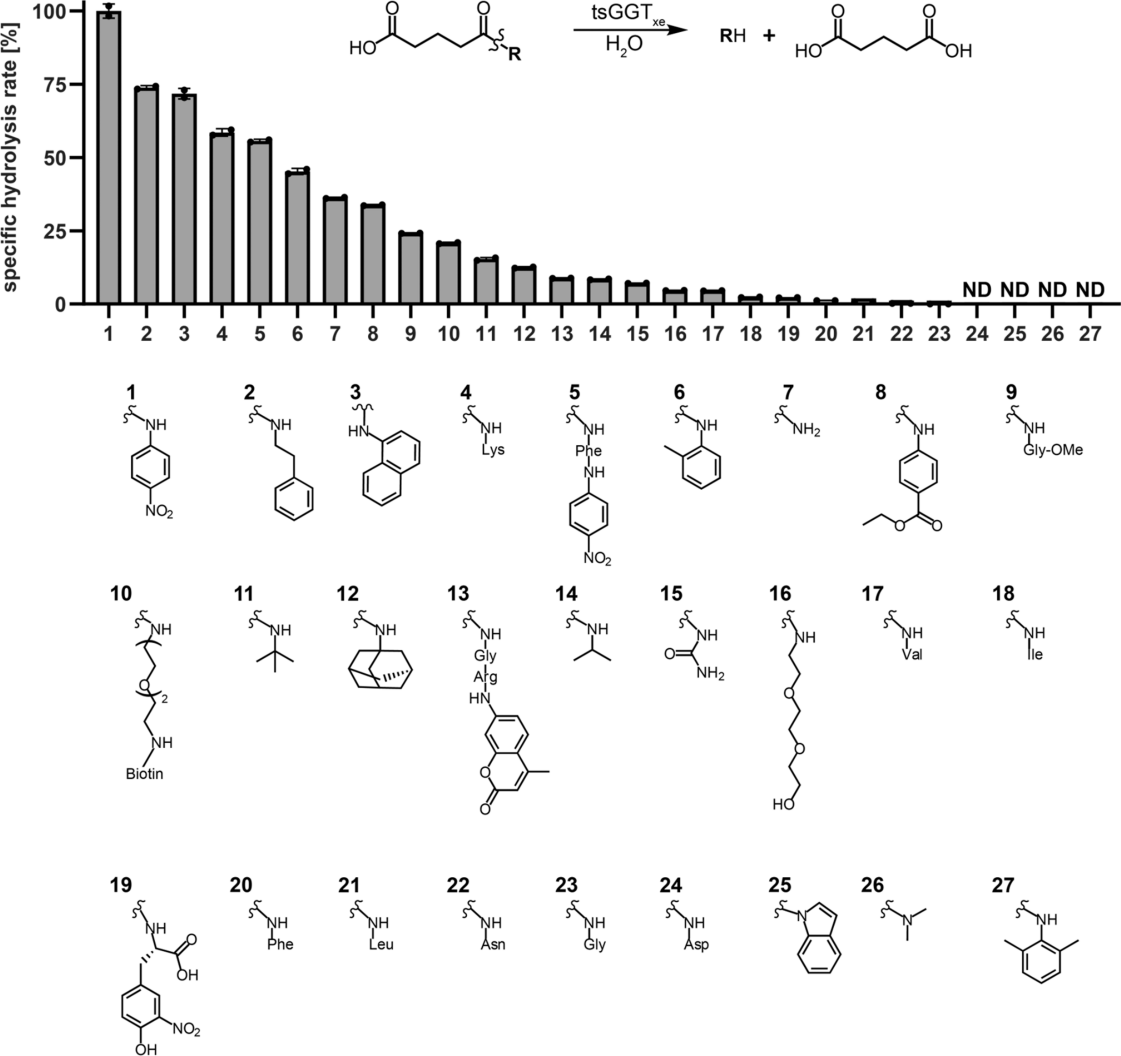
Characterizing the substrate promiscuity of tsGGT_xe_ with a GTA-conjugate library. Substrates are ordered according to the initial hydrolysis rate. Rates are normalized to the rate of GTA-pNA (**1**) (100% = 163 μM s^–1^ with 1 mg protein). All amino acid-containing conjugates (**4**, **5**, **9**, **13**, and **17**–**24**) were supplied in the l-amino acid configuration. ND: Below detection limit. All measurements were performed in duplicate. Data are from Figure S3.

The library includes molecules with sterically demanding cargoes (**6**, **11**, **12**, **14**, **26**, **27**), aromatic dyes (**1**, **5**, **13**), metabolites (**7**, **15**, **25**), and a set of canonical (**4**, **17**, **18**, **20**, **21**, **22**, **23**, **24**) and noncanonical amino acids (**9**, **19**) (Figure 2). To facilitate and standardize the recording of hydrolysis activities, we developed a quantitative assay based on the release of glutaric acid, which is the common release product in the hydrolysis reactions of all GTA-conjugates. For quantification, we used column-free inflow injection and mass spectrometry, more specifically we developed a selected reaction monitoring (SRM) method with quantification by comparison to a stable isotope labeled d4-glutaric acid (2,2,4,4-D4) variant (Figure S2). By comparing the initial velocities obtained for different cargo-vector conjugates, we could observe that tsGGT_xe_ exhibits distinct substrate preferences (Figures 2 and S3).

The highest activities were observed for conjugates with aromatic cargo molecules. Among the eight substrates with the highest initial hydrolysis rate, six cargo molecules contained an aromatic moiety such as p-nitroaniline (**1**), phenylethylamine (**2**), 1-naphthylamine (**3**), l-phenylalanin-4-nitronaniline (**5**), 2-methylaniline (**6**) and benzocaine (**8**). High activity was also observed for GTA-lysine (**4**) and glutaramate (**7**), the vector-cargo conjugate that is most similar to glutamine. Sterically more demanding conjugates containing cargo molecules such as isopropyl amine (**14**), *tert*-butylamine (**11**), and amantadine (**12**) were hydrolyzed with initial reaction rates still reaching 8–16% of the maximum rate observed in the screen, which was obtained for GTA-p-nitroanilide (**1**). In contrast, cargo-vector conjugates with cargo molecules that were linked via a tertiary amide, such as dimethylamine (**26**) and indole (**25**), were not processed by tsGGT_xe_ at all. To our surprise, the high enzymatic activity that was observed for 2-methylaniline (**6**), was completely lost in the case of 2,6-dimethylanilide (**27**), which only differs by one methyl group in the ortho-position of the aniline ring. Strikingly, GTA-conjugates of canonical and noncanonical amino acids that were linked via the α-amino group such as l-valine (**17**), l-isoleucine (**18**), 3-nitrotyrosine (**19**), l-phenylalanine (**20**), l-leucine (**21**), l-asparagine (**22**), l-glycine (**23**) and l-aspartate (**24**), showed consistently low hydrolysis rates. There were only two exceptions for glutaryl-amino acid cargoes for which high activities were observed, l-lysine (**4**) and l-glycine-methylester (**9**). Noteworthy, blocking the carboxylic acid group of l-glycine via methylester (**9**) resulted in significantly increased hydrolysis activity compared to the nonmodified l-glycine (**23**). In summary, the analysis of the diverse GTA-conjugate library helped us to confirm the overall broad substrate specificity of tsGGT_xe_, but it also points out curious groups of cargos for which the enzyme displays less activity, including a substantial number of amino acids.

### Improving tsGGT_xe_ Hydrolysis Activity Based on l-Leucine Auxotrophy Complementation

**Figure 3:**
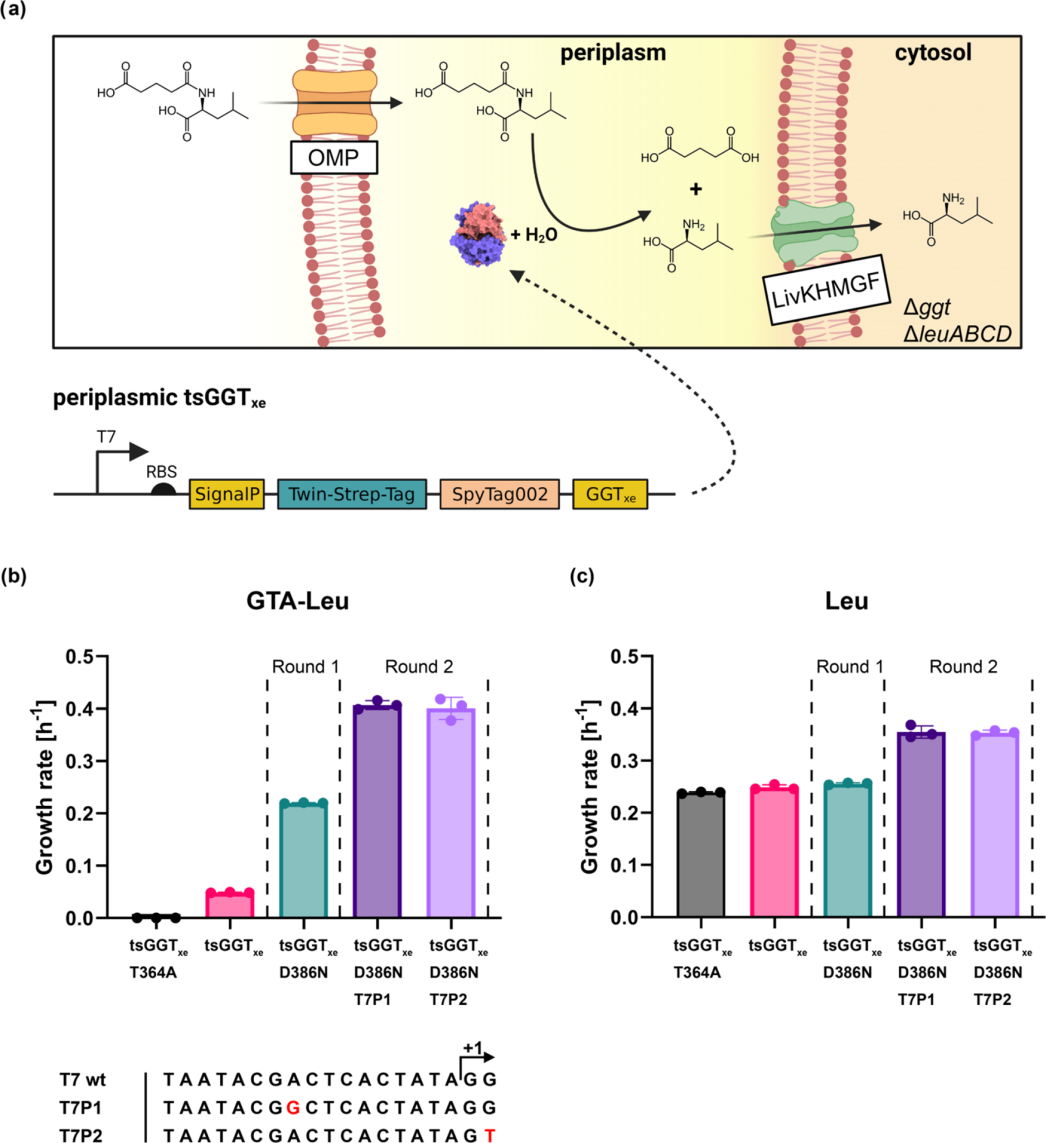
Directed evolution of periplasmic tsGGT_xe_ via l-leucine auxotrophy complementation. (a) Schematic illustration of tsGGT_xe_-dependent selection, based on l-leucine auxotrophy complementation. Periplasmic tsGGT_xe_ hydrolyzes GTA-Leu, and l-leucine is released, which leads to the complementation of l-leucine auxotrophy in XEc1 (MG1655 DE3 Δ*leuABCD* Δ*ggt*). (b) Specific growth rates of XEc1 cells expressing either the parental tsGGT_xe_ or the catalytically inactive tsGGT_xe__T364A variant with or without mutated transcriptional signals under selective growth conditions with 0.5 mM GTA-Leu as the only leucine source. (c) Same as (b), but specific growth rates were calculated for growth in nonselective medium with 0.5 mM l-leucine in the medium. OMP = outer membrane pore; LivKHMGF^31^ = *E. coli* ABC transporter of l-leucine.

Since canonical and noncanonical amino acids are interesting biological building blocks and therefore of high value for synthetic biology, we aimed to improve enzymatic activity on cargo molecules based on the amino acid backbone. To improve the catalytic activity of tsGGT_xe_ on GTA-amino acid conjugates, we established a growth-based selection system depending on the hydrolysis activity of tsGGT_xe_*in vivo*. To this end, we used an l-leucine auxotrophic *E. coli* strain^29^ (MG1655 DE3 Δ*leuABCD* Δ*ggt*, hereafter referred to as XEc1), which cannot grow in a defined medium (M9^30^ supplemented with 0.4% fructose) without an external source of l-leucine. As it is unclear whether GTA-Leu can cross the *E. coli* inner membrane, we directed the expression of tsGGT_xe_ to the periplasm by attaching the endogenous signal peptide of *Pn*GGT to the N-terminus of tsGGT_xe_ (Figure 3a) and thereby relocated the enzyme to the periplasm of XEc1 (Figure S4).

According to the experimental design, tsGGT_xe_ or its variants hydrolyze GTA-Leu in the periplasmic space and release enough l-leucine to complement the l-leucine auxotrophy of XEc1 (Figure 3a). In a control experiment, we found that the enzymatic activity of parent tsGGT_xe_ expressed in the periplasm is required and sufficient to complement the l-leucine auxotrophy: When XEc1 producing either active periplasmic tsGGT_xe_ or catalytically inactive tsGGT_xe_ (tsGGT_xe_ T364A) was grown in M9 medium supplemented with 0.4% fructose and 0.5 mM GTA-Leu growth was observed only with the catalytically active tsGGT_xe_ (Figures 3b and S5a). In contrast, similar growth under nonselective conditions (0.5 mM l-leucine instead of GTA-Leu) was obtained for both strains (Figures 3c and S5b). The difference in specific growth rates of tsGGT_xe_-producing strain under selective and nonselective conditions (selective: 0.05 h^–1^, nonselective: 0.25 h^–1^) suggested that selecting for tsGGT_xe_ variants with better catalytic performance for the hydrolysis of GTA-Leu could be possible.

In order to generate the required diversity for selection, we decided for a naïve library approach and used error prone PCR to generate a library of mutations into the small subunit of tsGGT_xe_ (library size: 1.25 million clones, average mutation rate of 10 individually isolated clones: 8.1 aa per 1000 aa changed) was used to transform strain XEc1. After plating on selective M9 medium supplemented with 0.4% fructose, 0.5 mM GTA-Leu, 50 μg mL^–1^ kanamycin, and 50 μM IPTG, we identified eight colonies that showed faster growth than cells producing the parental tsGGT_xe_. We therefore isolated the plasmids and identified the mutations via Sanger sequencing. Strikingly, all eight plasmids shared the D386N mutation (aspartate to asparagine). Two variants contained additional mutations which were silent: either H489H or N379N and G390G (Figure S6a). This strongly suggests that the D386N mutation is responsible for the altered growth on GTA-Leu. In order to confirm this assumption, we separately reinserted the D386N mutation into the original parent plasmid and used the resulting construct to transform XEc1. The resulting strain showed indeed an increased specific growth rate (0.22 h^–1^) compared to the parental variant (0.05 h^–1^), suggesting that this mutation is indeed responsible for improved GTA-Leu hydrolysis (Figure 3b).

In an attempt to potentially further improve GTA-Leu hydrolysis, we undertook another round of epPCR using the gene of the tsGGT_xe__D386N variant as the template (library size: 1.2 million clones; average mutation rate of 10 individually isolated clones: 8.1 aa per 1000 aa changed) and selected XEc1 transformants on a more stringent selective medium containing only 0.25 mM GTA-Leu. Five clones that were able to grow faster than XEc1 expressing the gene for tsGGT_xe__D386N were isolated, and the corresponding sections of the isolated plasmids were sequenced. While none of these plasmids contained any new mutation in the tsGGT_xe__D386N coding sequence that would lead to a mutation on primary sequence level, all contained changes in the promoter region and four plasmids contained additional silent mutations in the tsGGT_xe__D386N gene sequence (Figure S6b). Three identical plasmids showed the change A to G in position 8 of the T7 promoter (change referred to as T7P1) and two more plasmids showed a change G to T in nucleotide two of the predicted mRNA (change referred to as T7P2). Interestingly, both mutations had been described in earlier publications^32,33^ and were associated with reduced transcriptional activity of the T7 promoter. As the mutations in the tsGGT_xe__D386N coding sequence were all silent and literature suggested measurable effects for the changes in the noncoding regions, we focused on the latter and separately introduced changes T7P1 and T7P2 into the parental plasmid. Transformants with the plasmids containing either of these changes indeed showed increased growth rates (Figure 3b) as well as increased growth yields (Figure S5). We therefore conclude that growth in the second round of directed evolution was no longer limited by the enzymatic activity of tsGGT_xe__D386N but rather by the metabolic burden associated with the increased gene expression of a periplasmic protein.

To complete this part of the investigation, we tested whether Asn at position 386 indeed gave the best growth on a selected medium with GTA-Leu. We constructed an NNK-site saturation library at position 386 and sequenced 45 of the largest colonies that had appeared after plating XEc1 transformants on a selective medium containing 0.5 mM GTA-Leu. Sequence analysis revealed 14 different amino acids (D, L, Y, Q, H, V, W, T, C, A, G, R, N, and S) at position 386 in these 45 plasmids, including the original Asp. However, there were clear differences in the frequency with which some amino acids at position 386 were found in this set, with Ser and Asn as the most frequent substitutions, suggesting that these two mutations favor growth in selective medium (Figure S6c).

**Figure 4:**
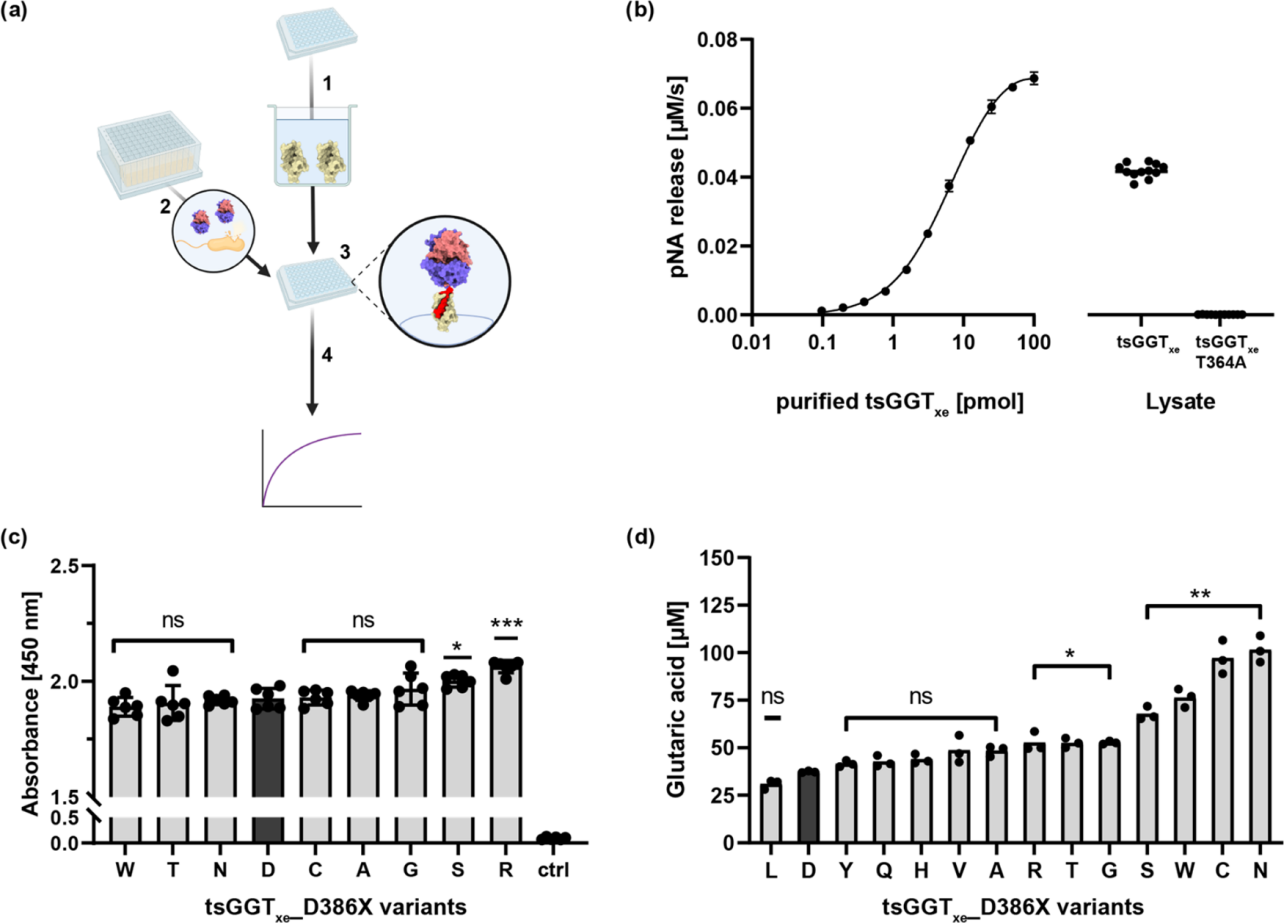
*In vitro* characterization of tsGGT_xe_ variants using an enzyme immobilization-based activity assay. (a) Schematic illustration of the secondary enzyme activity assay based on SpyCatcher/SpyTag-mediated enzyme immobilization and MS/MS. (1) A 96-well ELISA plate was coated with a fixed amount of purified SpyCatcher002 protein, (2) clones from selection were grown in ZYM-5052 autoinduction medium,^35^ chemically lysed and the clarified lysate transferred (3) into the SpyCatcher002 coated ELISA plate. After SpyCatcher002-SpyTag002-mediated enzyme immobilization and several washing steps, the enzymatic activity of the immobilized tsGGT_xe_ variants was determined. (b) Concentration-dependent immobilization of purified tsGGT_xe_ (0.05–100 pmol/well) in 96-well MTP via SpyCatcher002 (24 pmol/well). Enzymatic activity of immobilized tsGGT_xe_ was measured via initial velocity of hydrolysis using colorimetric GTA-pNA (absorbance at 405 nm). Additionally, lysates of XEc1 expressing tsGGT_xe_ or tsGGT_xe__T364A were prepared and immobilized (*n* = 12), and GTA-pNA was used to measure activity (rate of 0.042 ± 0.002 μM/s). (c) Comparison of immobilization levels of tsGGT_xe__D386X variants derived from lysates (*n* = 6) via sandwich-like ELISA using an anti-Streptag-HRP antibody conjugate. The product of the colorimetric HRP substrate was measured via the absorbance at 450 nm. Ctrl = no lysate added, only lysis buffer. A one-way ANOVA with Dunnett’s multiple comparisons post hoc test was performed to compare immobilization levels of tsGGT_xe__D386X variants with the parental tsGGT_xe_ variant (ns, not significant (adj. *p*-value ≥0.033)); * (adj. *p*-value <0.033); ** (adj. *p*-value <0.002); *** (adj. *p*-value <0.001). (d) Enzymatic activities of tsGGT_xe__D386X hits derived from the D386 site saturation library were immobilized and compared via end point measurement of released glutaric acid through MS/MS after 17 h of incubation using 1 mM GTA-Leu at 37 °C. Measurements were performed in triplicate. A one-way ANOVA with Dunnett’s multiple comparisons post hoc test was performed to compare the glutaric acid levels of each variant with the wildtype (D386). (ns, not significant (adj. *p*-value ≥0.01)); * (adj. *p*-value <0.01); ** (adj. *p*-value ≤0.001).

So far, all observations were based on growth in selective medium, and we next investigated the enzymatic activity of the different tsGGT_xe__D386X variants. For this, we exploited the capacity of immobilized SpyCatcher protein to covalently bind Spy-tagged proteins as suggested before,^34^ and then used hydrolysis of chromogenic GTA-pNA (**1**) or MS/MS to track the activity of retained and presumably purified tsGGT_xe_ variants (Figure 4a). We obtained the binding partner for the SpyTag, SpyCatcher002, by affinity chromatography (Figure S7), and confirmed the isopeptide formation between purified SpyCatcher002 and purified tsGGT_xe_ using SDS-PAGE (Figure S8). This capturing step could also be repeated in microtiter plate format, and we found the immobilization of tsGGT_xe_ to be concentration-dependent (Figure 4b): we coated wells of a 96-well ELISA plate using 4 μg mL^–1^ (24 pmol/well) of purified SpyCatcher002, and then added different amounts of purified tsGGT_xe_ ranging from 0.05 to 100 pmol to the wells, followed by incubation for 90 min. After the sample was washed, a GTA-pNA (**1**) substrate solution was added, and total enzyme activities based on initial rates for the development of the chromogenic pNA were calculated. The analysis suggests that the immobilized SpyCatcher002 proteins in the wells are saturated when around 50 pmol per well of tsGGT_xe_ are provided. When we repeated this experiment but used tsGGT_xe_ in cell lysate instead of purified tsGGT_xe_, we found the activity that could be obtained per well 30% lower, though highly reproducible (Figure 4b), which suggested to us that the immobilization process had still reached saturation under these conditions but was less efficient when using protein from cell lysates. In support of this, we produced cell lysates from nine different tsGGT_xe__D386X variants, applied them to adsorption in coated microtiter plates, and then used an antibody against the N-terminal Twin-streptag of the tsGGT_xe__D386X variants for quantification of the amount of adsorbed protein. We found 7 variants to display very similar amounts of adsorbed proteins (Figure 4c), and we concluded that the measurements in this secondary assay format reflected specific activities with little influence of expression levels.

Next, we used the developed assay to characterize the different tsGGT_xe__D386X variants. We prepared lysates of all 14 tsGGT_xe__D386X variants, immobilized the protein variants, and incubated them with 1 mM GTA-Leu for 18 h and then used the previously established MS/MS method to quantify the concentration of glutaric acid. The measurements for four out of 14 tested variants (with N, C, W, or S in position 386) showed indeed significantly increased glutaric acid concentrations compared with the parental version tsGGT_xe_ (Figure 4d). Interestingly, the highest glutaric acid concentration was measured with the previously identified D386N variant. On the other hand, the remaining 10 variants (L, D, Y, Q, H, V, A, R, T, or G in position 386) showed no significant or only small increases in the low final glutaric acid concentration among each other. As tsGGT_xe__D386N was the most active variant for hydrolysis of GTA-Leu, we therefore continued with this variant.

### Characterizing tsGGT_xe__D386N on Vector-Amino Acid Conjugates

**Figure 5:**
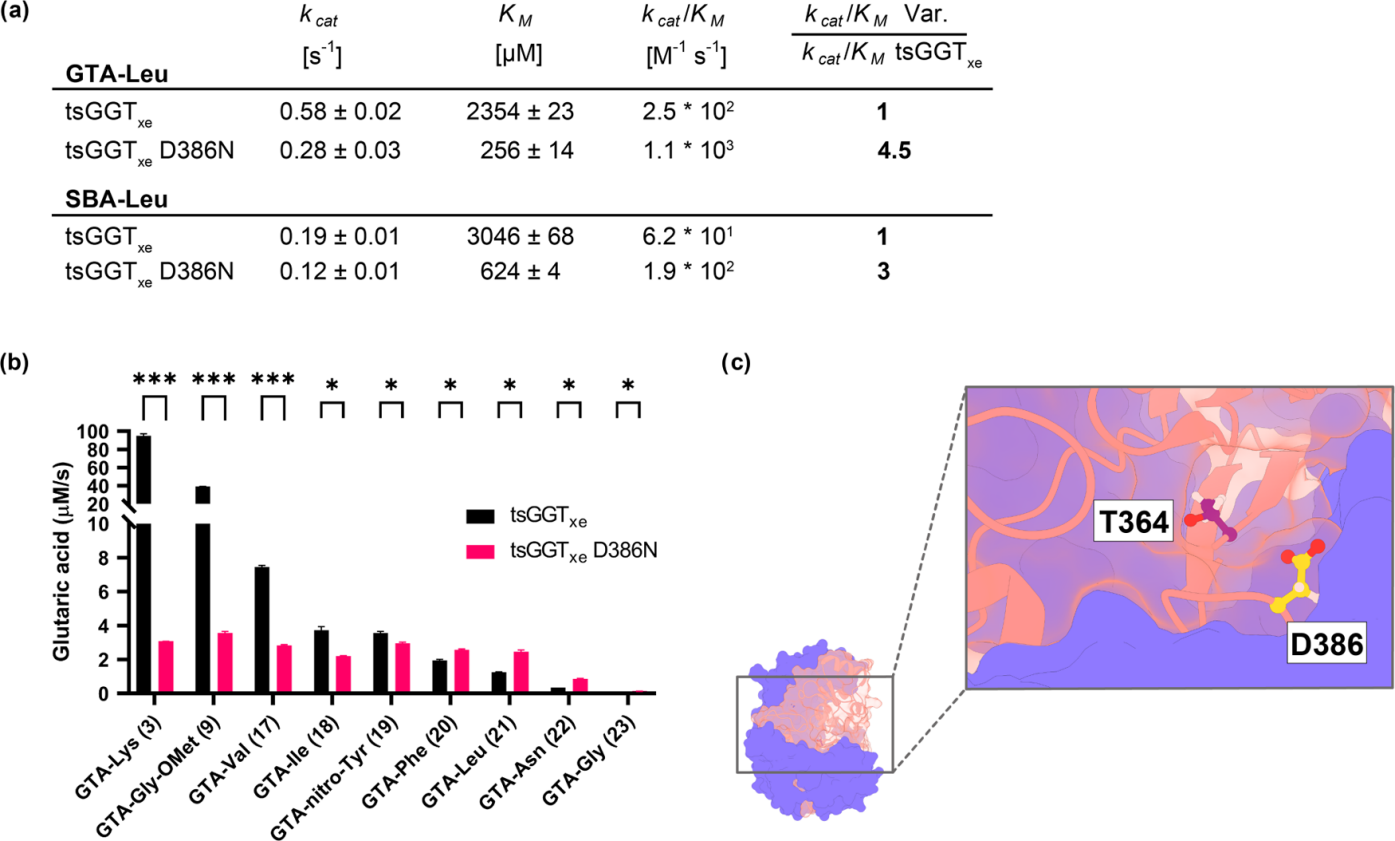
Kinetic characterization of tsGGT_xe__D386N. (a) Michaelis–Menten parameters of purified protein for tsGGT_xe_ and tsGGT_xe__D386N using GTA-Leu or SBA-Leu as substrates. (b) Comparison of initial hydrolysis rates (as determined by glutaric acid release by MS/MS) of tsGGT_xe_ and tsGGT_xe__D386N with different GTA-amino acid conjugates. All measurements were performed in duplicates. An unpaired *t* test was used to compare the hydrolysis rates between tsGGT_xe_ and tsGGT_xe__D386N (ns, not significant (*p*-value ≥0.05)); * (*p*-value <0.05); ** (*p*-value <0.005); *** (*p*-value <0.0005), Holm–Sidak’s multiple comparisons test. (c) Surface-cartoon illustration of the *P. nitroreducens* GGT (PDB: 7D9E) crystal structure.^25^ The large subunit is illustrated as a blue surface, and the small subunit is illustrated as an orange cartoon structure. The catalytic threonine at position 364 (purple residue) is in close proximity (distance: ∼7 Å) to the aspartate at position 386 (yellow residue), which is located right at the entrance to the substrate binding site.

Finally, we investigated if the increased enzymatic activity for tsGGT_xe__D386N is specific for GTA-Leu only or if other vector-amino acid conjugates would also get hydrolyzed with increased rates. First, we tested the influence of the vector and determined the kinetic parameters for the enzyme using either GTA-Leu or SBA-Leu as substrates by enzymatic detection of branched-chain amino acids. This assay couples product formation (l-leucine) to a colorimetric readout that can be measured at 450 nm. We therefore incubated purified tsGGT_xe_ or tsGGT_xe__D386N with an excess of different concentrations of either GTA-Leu or SBA-Leu, recorded the initial hydrolysis rates, and fitted the resulting data to a Michaelis–Menten kinetic (Figure S9). For GTA-Leu, the data indicated that tsGGT_xe__D386N showed a roughly 9-fold decreased *K*_*M*_ and a roughly 2.3-fold decreased *k*_cat_, resulting in an increase of 4.5-fold in catalytic efficiency compared to tsGGT_xe_ (Figure 5a). For SBA-Leu, we observed a very similar overall effect of the D386N mutation: while the determined *K*_M_ value was slightly higher and the *k*_cat_ value was slightly lower than for GTA-Leu as the substrate, the mutated enzyme still showed a 3.0-fold improvement in catalytic efficiency.

Next, we examined whether the increased enzymatic activity of tsGGT_xe__D386N also extends to amino acid cargoes other than Leu when GTA is used as the vector. We therefore determined the initial hydrolysis rates for the nine previously measured GTA-amino acid conjugates via MS/MS (Figure 5b). The direct comparison of the hydrolysis rates obtained with tsGGT_xe_ or tsGGT_xe__D386N clearly indicated that the D386N mutation has a broad, but difficult-to-predict influence: For some GTA-amino acid conjugates, increased hydrolysis activity was observed (**20, 21, 22, 23**), but for others (**3, 9, 17, 18, 19**), the initial hydrolysis rate under screening conditions was significantly lower compared to that of the parental tsGGT_xe_. This reduced reaction rate was most prominent for GTA-Lys (**3**) and GTA-Gly-OMet (**9**).

To find a potential explanation for the effect of the D386N mutation, we inspected the crystal structure of *Pseudomonas nitroreducens* GGT (PDB: 7D9E),^25^ which differs from GGT_xe_ by the absence of the N-terminal affinity tags and the D406N mutation and thus is still a good model for tsGGT_xe__D386N. In this structure, the side chain of residue D386 is located in close proximity (7 Å) to the catalytic nucleophile T364 and is oriented toward the substrate binding site (Figure 5c). It is therefore very likely that D386 directly interacts with the amino acid cargo molecule. Since the change from aspartate to asparagine is essentially the removal of a negative charge close to the substrate binding site, it seemed likely that the mutant protein got rid of the electrostatic repulsion between aspartate 386 and the carboxylic acid moiety of the amino acid substrate. In support of this hypothesis, the hydrolysis of GTA-Gly (**23**) was substantially slower than the hydrolysis of GTA-Gly-OMet (**9**).

## Discussion

We set out to extensively characterize the substrate promiscuity of tsGGT_xe_ in view of its role as a potentially generic cargo release enzyme in a synthetic import system. We made use of the good comparability of SBA-conjugates with GTA-conjugates and the good availability of different examples for the latter, which allowed us to perform an in-depth substrate tolerance characterization of tsGGT_xe_. Our findings suggest that conjugates containing an aromatic moiety close to the linking amide bond are preferred substrates. By using a set of ortho-substituted (mono- or disubstituted) as well as unsubstituted aniline-based cargo molecules, we could characterize the ability of tsGGT_xe_ to deal with sterically demanding moieties: A single methyl group at the ortho-position of aniline is well tolerated, but if both ortho positions are substituted, the enzymatic activity is lost. We hypothesize that the additional methyl group clashes with one of the amino acid residues located in the vicinity of the catalytic nucleophile of tsGGT_xe_. Also, tsGGT_xe_ does not process tertiary amides and shows only small hydrolysis activities on most proteogenic amino acid conjugates tested in this screen, except l-lysine. Some of the here described substrate preferences can also be observed in a study conducted on *P. nitroreducens* GGT and glutamyl-conjugates by Imaoka et al.,^36^ although different substrate concentrations and reaction conditions make a direct comparison difficult. In general, the fact that tsGGT_xe_ showed hydrolysis activity above background level on 23 out of 27 tested GTA-conjugates clearly demonstrates the overall broad substrate tolerance of the enzyme.

Since the import of noncanonical amino acids might be a promising area of application for the synthetic import system, we investigated in more detail the vector-amino acid conjugate hydrolysis activity of tsGGT_xe_, which was on the lower end of the observed rate spectrum. We used directed evolution based on auxotrophy complementation to identify an enzyme variant with a higher hydrolysis rate. Although the first round of evolution yielded a tsGGT_xe_ variant (D386N) with improved catalytic efficiency for the hydrolysis reaction of GTA-Leu, the second round of evolution did not increase the activity further, but seemed to lead to reduced gene expression,^32,33^ suggesting that the metabolic burden of the periplasmic expression had become the easiest parameter to improve for the cell to escape the selective pressure. The *in vitro* characterization of tsGGT_xe__D386N has shown that residue D386 plays a crucial role in the enzymatic hydrolysis of GTA- and SBA-amino acid conjugates and most likely does so by interfering with the negatively charged carboxylic acid moiety of the amino acid substrate. Here, it is interesting that this effect can apparently be compensated by adding a methyl group to the carboxyl group of the amino acid. Our results are consistent with a previous publication postulating that the negative charge of residue D386 may be important for recognition of the acceptor substrate during catalysis.^25^ In addition, we demonstrated that tsGGT_xe__D386N also exhibited enhanced kinetic properties on SBA-Leu, supporting our notion that GTA-conjugates are an appropriate choice for characterizing the substrate promiscuity of tsGGT_xe_. Overall, we conclude that tsGGT_xe_ indeed has a very relaxed substrate specificity, making it a good candidate for a universal cargo release module in a synthetic transport system.

## Materials and Methods

### Materials and Chemicals

If not stated differently, chemicals were obtained from Sigma-Aldrich (now Merck, Darmstadt, Germany), AppliChem (Darmstadt, Germany), DNA isolation and purification kits from Zymo Research (Irvine), and enzymes and Q5 DNA polymerases from New England Biolabs (Ipswich). DNA fragments were obtained from IDT (Coralville) or Microsynth (Balgach, Switzerland). Custom-synthesized glutaryl or sulfobutanoyl conjugates were obtained from ChemSpace (New Jersey). Sanger sequencing analysis was performed by Microsynth (Balgach, Switzerland). Electrocompetent cells were obtained from Lucigen (Middleton) or Merck (Darmstadt, Germany). *E. coli* BL21 DE3 was obtained from Novagen (now Merck, Darmstadt, Germany). LB medium and LB-agar plates were prepared using Lysogeny Broth obtained from Becton Dickinson (New Jersey). 96-Well ELISA plates “Nunc MaxiSorp 96-well plate” were obtained from Thermo Fisher Scientific (Waltham). HPLC solvents were obtained from Merck (LC-MS grade, LiChrosolv).

### Construction of pDM_tsGGT_xe_ and pDM_Δ*S*ignalP_tsGGT_xe_

If not stated differently, all PCR products were analyzed using agarose gel electrophoresis and extracted using a Zymoclean Gel DNA Recovery Kit (Zymo Research). The gene encoding for *Pseudomonas nitroreducens* GGT lacking the signal peptide (ΔN24) (Uniprot entry: E0D5C2) was obtained as a double-stranded DNA gBlock (IDT DNA). The residues of *Pn*GGT (Uniprot entry: E0D5C2) are annotated according to Hibi et al.^24^ and are identical, except for T506P. The same DNA gBlock also encoded a synthetic RBS (TIR:78636) and a SpyTag002-GSGESGEL directly fused to ΔN24_*Pn*GGT_D406N (*Pn*GGT lacking the signal peptide (N24) and containing the D406N amino acid substitution). The obtained double-stranded DNA gBlock was ligated with the linearized pGFP^37^ plasmid (based on pSEVA261) via Gibson Assembly.^38^ Beforehand, the linearized pGFP was generated via PCR using primers oDAM001, oDAM002 (Table S1) and Q5 Polymerase (New England Biolabs). The protocol yielded the intermediate plasmid pDM_Spy_GGT_xe_, which was linearized by PCR using primers oDAM001, oDAM018. The linearized fragment was subsequently ligated with gBlocks encoding either an N-terminal Twin-streptag or the *P. nitroreducens* signal peptide (ΔN24) fused to a Twin-streptag, yielding plasmids pDM_Δ*S*ignalP_tsGGT_xe_ or pDM_tsGGT_xe_, respectively.

### Error Prone Library Generation Targeting the Small Subunit of tsGGT_xe_

Error prone PCR was conducted on the small subunit of tsGGT_xe_ with primers oDAM102 and oDAM105 (Table S1) and plasmid pDM_tsGGT_xe_ as the template over 30 cycles using a Pfu DNA polymerase mutant lacking exonuclease activity.^39^ To obtain the linearized vector, a PCR was conducted using Q5 Polymerase (New England Biolabs) and primers oDAM103 and oDAM104 and pDM_tsGGT_xe_ as the template. The purified insert fragment was ligated with the purified vector via Gibson Assembly.^38^ Obtained plasmid libraries were used to transform E. cloni 10G Elite electrocompetent cells (Lucigen) or SIG10 Ultra electrocompetent cells (Sigma-Aldrich). Cells were recovered in 1 mL of LB medium (Lysogeny Broth, Becton Dickinson) at 37 °C and 220 rpm for 1 h. Afterward, a serial dilution of the cells on LB-agar plates containing 50 μg mL^–1^ kanamycin was used to calculate the theoretical library size and estimate the library diversity.

### Site Saturation Mutagenesis of Residue 386

The site saturation NNK-library of residue D386 was constructed by PCR with a mutagenic primer. Specifically, plasmid pDM_tsGGT_xe_ was used as the template, and primers oDAM033 (containing the degenerated NNK-sequence) and oDAM056 were used for amplification. A second fragment was generated by PCR with primers oDAM034 and oDAM055 and pDM_tsGGT_xe_ as the template. The PCRs were conducted with Q5 Polymerase (New England Biolabs). The purified insert fragment was ligated with the purified vector via Gibson Assembly.^38^

### Genome Engineering of XEc1 (*E. coli* MG1655 DE3 ΔleuABCD Δggt)

*E. coli* MG1655 Δ*leuABCD* was obtained from V. Pezo (Genoscope, Évry, France). The gene encoding for GGT (*ggt*) was removed from the genome using the λ red recombineering system.^40^ In short, the kanamycin resistance gene from plasmid pKD13 was amplified using primers ERR057F and ERR057R (Table S1), which contain 50 bp of the *ggt* flanking regions as overhangs. A culture of *E. coli* MG1655 Δ*leuABCD* transformed with pKD46 was grown overnight in LB medium supplemented with 35 μg mL^–1^ carbenicillin at 30 °C with no shaking. Afterward, induced with 15 mM l-arabinose for 2 h at 30 °C, 220 rpm for the expression of the λ red recombination system. Cells were then transformed with ∼500 ng of PCR product and plated on LB-agar supplemented with 50 μg mL^–1^ kanamycin to select for positive transformants. Successful substitution of the *ggt* gene for the kanamycin cassette was confirmed via colony PCR using primers ERR058F and ERR058R (Table S1). For the removal of the kanamycin resistance gene from the genome, *E. coli* MG1655 Δ*leuABCD* ggt::kanR was transformed with pCP20 and 8 clones were restreaked on LB-agar supplemented with 35 μg mL^–1^ carbenicillin at 30 °C. Colonies were then restreaked on LB-agar only at 37 °C, until positive clones showed no growth on either LB + 50 μg mL^–1^ kanamycin or LB + 35 μg mL^–1^ carbenicillin. Successful removal of the kanamycin cassette from the genome was confirmed via colony PCR using primers ERR058F and ERR058R.

In order to genomically introduce the gene encoding for T7 RNA polymerase under *lacUV5* into the *E. coli* MG1655 Δ*leuABCD* Δggt strain, the λDE3 Lysogenization Kit (Novagen) was used according to the manufacturer’s protocol. The genome of the obtained strain was confirmed using the next-generation sequencing service from Novogene (Beijing, China).

### Cell Fractionation to Determine Cellular Localization of tsGGT_xe_ and Δ*S*ignalP- tsGGT_xe_

To determine cellular localization of tsGGT_xe_ activity *E. coli* cells were fractionated into periplasmic and cytosolic fractions by combining digestion of the cell wall with osmotic shock, similar to the approach described by Kuenzl et al.^11^ Therefore, XEc1 cells harboring either pDM_tsGGT_xe_ or pDM_ΔsignalP-tsGGT_xe_ were grown (220 rpm, 37 °C) in LB medium supplemented with 50 μg mL^–1^ kanamycin until an OD_600_ of 0.4–0.5 was reached. Gene expression was induced by setting the final concentration of IPTG to 0.5 mM and further incubated at 22 °C, 200 rpm for 4 h. Afterward, 2 mL of each culture was spun down (1500 rcf, RT for 15 min) and the supernatant was removed. To release periplasmic fraction, the pellets were gently resuspended in 100 μL of periplasting buffer (20% (w/v) sucrose, 1 mM EDTA, 30000 U mL^–1^ lysozyme, and 200 mM Tris/HCl pH 7.5) and incubated at RT for 5 min. Next, 100 μL of cold water was added and gently mixed, followed by incubation on ice for another 5 min. To isolate the periplasmic fraction, the partially lysed cells were centrifuged at 2500 rcf and RT for 2 min, and the supernatant was collected in a fresh tube. The remaining pellet was again resuspended in 100 μL of periplasting buffer, incubated at RT for 5 min, then centrifuged at 2500 rcf, RT for 2 min after which the supernatant was removed. The pellet was resuspended in 200 μL of lysis buffer (50 mM KCl, 1 mM EDTA, 0.1% (w/v) sodium deoxycholate, 5 mM MgCl_2_, 300 Kunitz mL^–1^ Dnase I, and 10 mM Tris-HCl pH 7.5) and incubated at RT for 5 min. The sample was centrifuged at 2000 rcf and RT for 5 min, and the supernatant was collected in a clean tube as cytoplasmic fraction. Purity of the fractions was determined by measuring the activity of the periplasmic enzyme alkaline phosphatase and the cytoplasmic enzyme β-glucuronidase as described by Kuenzl et al.^11^ The tsGGT_xe_ activity in the periplasmic and the cytosolic fractions were measured via hydrolysis of GTA-p-nitroanilide, leading to release of *p*-nitroaniline at 37 °C. For this, 5 μL of periplasmic or cytosolic fraction was incubated in a total volume of 250 μL of 10 mM ammonium bicarbonate (NH_4_HCO_3_) in dH_2_O containing 1 mM GTA-pNA and absorbance at 405 nm was measured continuously with an MTP reader (Tecan infinite 200Pro) heated to 37 °C. For each progress curve, the initial velocity was calculated and used to calculate relative activities in the cytosolic and periplasmic fraction.

### Growth Selection on GTA-Leu

In order to select for increased tsGGT_xe_ hydrolysis activity on GTA-Leu, the electrocompetent selection strain XEc1 was transformed with pDM_tsGGT_xe_ or variants thereof. After 1 h of recovery in LB medium at 37 °C, cells were spun down at 4000 rcf for 5 min using a centrifuge. The supernatant was removed, and cells were resuspended in 1× M9 salts. The washing step was repeated twice. Afterward, cells were spread onto M9 agar plates containing 0.4% fructose, 50 μg mL^–1^ kanamycin, 50 μM Isopropyl-β-d-thiogalactopyranosid (IPTG), and GTA-Leu as indicated in the main text. Plates were incubated at 37 °C, and colonies that appeared earlier than those on a control plate, containing XEc1 [pDM_tsGGT_xe_] or [pDM_tsGGT_xe__D386N], were picked and verified by restreaking on fresh selection plates.

### Characterization of tsGGT_xe_ Variants by Growth

To quantify the influence of tsGGT_xe_ and variants thereof on growth under selective and nonselective conditions, a microtiter plate-based growth assay was used. XEc1 harboring pDM_tsGGT_xe_ or variants thereof were inoculated in 10 mL of M9 medium with 0.4% fructose, supplemented with TES^41^ (trace element solution), 50 μg mL^–1^ kanamycin, 50 μM IPTG, and 0.5 mM l-leucine and grown in a 50 mL shake flask at 37 °C, 200 rpm. 1 mL of the fully grown culture was then spun down (4000 rcf, RT for 10 min), and the supernatant was removed. The pellet was resuspended with 1 mL of 1× M9 salts and spun down, as described above. The washing step was repeated twice, and the cells were finally resuspended in 1 mL of 1× M9 salts. The optical density was determined (OD_600_) and diluted in selective or nonselective M9 minimal medium to OD_600_ = 0.05. For selective conditions, 100 μL of M9 medium with 0.4% fructose, supplemented with TES^41^ (trace element solution), 50 μg mL^–1^ kanamycin, 50 μM IPTG, and 0.5 mM GTA-Leu in a 96-well MTP were used. For nonselective growth, GTA-Leu was replaced with l-leucine. Growth was measured by tracking OD_600_ over time at 37 °C with constant orbital shaking (6 mm amplitude) in a microtiter plate reader (Tecan Infinite 200Pro).

### Enzyme Activity Assay Based on Glutaric Acid Quantification Using MS/MS

Enzyme activity with GTA-conjugates was determined by measuring the concentration of released glutaric acid using MS/MS. Free glutaric acid was quantified using a selected reaction monitoring (SRM) method, tracking the transition of the negatively charged molecule ion (*m*/*z* = 131) to the decarboxylated fragment (*m*/*z* = 87) in negative mode using a QTRAP4000 MS/MS (AB Sciex LLC, Framingham). To quantify the reaction product, a fixed concentration of isotopically labeled d4-glutaric acid (2,2,4,4-D4, Cambridge Isotope Laboratories, Tewksbury) was added to the reaction mixture and used for normalization. The integrated signal obtained from the product transition *m*/*z* = 131 → *m*/*z* = 87 was normalized with the corresponding transition of the stable isotope standard *m*/*z* = 135 → *m*/*z* = 91 and compared to a calibration curve. In order to track the reaction progress, a 1 mL sample containing typically 1 mM GTA-conjugate, 200 μM d4-glutaric acid (2,2,4,4-D4), and 100 nM purified tsGGT_xe_ or variants thereof in 10 mM aqueous NH_4_HCO_3_ (LC-MS grade, Merck) adjusted to pH 7.4 with formic acid was incubated at 37 °C in an autosampler unit of an HPLC (Agilent Series 1200 autosampler). The outlet tubing of the autosampler unit was directly connected to the interface of the QTRAP4000 MS/MS device without using an HPLC column. The same sample was injected several times over the time course of 4 h (10 μL/injection) directly into mobile phase (700 μL min^–1^, 50:50 v/v acetonitrile/water) and was subsequently analyzed via MS/MS. The obtained raw data file “.wiff” was converted with msConvert^42^ (ProteoWizard, Version: 3.0.19217-f7f3a630b) into rich “.txt” files and MS/MS data extracted using a dedicated python script (Supplementary Code) or accessed through: https://git.bsse.ethz.ch/BPL_Panke/dam_msms_gta.

The python script yields a .csv spreadsheet, containing sample name, measuring time point in seconds relative to start time and integrated peak area of analyte and internal standard. The following QTRAP4000 settings were used: IonSpray Voltage (IS): −4500, Temperature (TEM): 750, Ion Source Gas 1 and 2 (GS1, GS2): 30, Collision Gas (CAD): 4, Declustering Potential (DP): −65 and Collision Energy (CE): −18.

### Immobilization of tsGGT_xe_ or Variants Thereof Using SpyCatcher Plates

First, plates were coated with the SpyCatcher002 protein. For this, a Nunc MaxiSorp 96-well plate (ThermoFisher Scientific) was coated with purified SpyCatcher002 protein to immobilize tsGGT_xe_ or variants thereof from crude cell lysate via SpyCatcher-SpyTag-mediated isopeptide formation.^43,44^ To each well, we added 100 μL of 50 mM phosphate buffer at pH 5.8, containing 4 μg mL^–1^ (24 pmol/well) SpyCatcher002 and incubated the plate for 18 h at 4 °C. Afterward, the liquid was discarded and each well washed three times with 150 μL of blocking solution (10 mM NH_4_HCO_3_, pH 7.4), containing 1% bovine serum albumin (BSA, Sigma-Aldrich). After washing, 250 μL of blocking solution was added per well, and the plate was incubated for 2 h at 37 °C. The blocking solution was discarded, and the wells were washed three times using 150 μL of blocking solution per well. After this, the “SpyCatcher plate” was ready for immediate use.

Samples containing SpyTag-labeled protein variants were prepared as follows: Overnight cultures of strains (based on *E. coli* strains BL21 DE3 or XEc1) harboring pDM_tsGGT_xe_ or variants thereof were grown at 37 °C, 220 rpm in a 96-deep well plate using 1 mL of ZYM-5052 autoinduction medium^35^ per well for 18 h at 37 °C with shaking at 220 rpm. After centrifugation at 4,000 rcf for 10 min, the supernatant was discarded and 300 μL of lysis buffer^45^ (6000 U mL^–1^ lysozyme (Merck), 30 μM polymyxin B sulfate (Merck) and 2% TritionX-100 (Merck) in 100 mM sodium phosphate buffer at pH 7.4) was added to each well. The pellet was resuspended in lysis buffer, and the plate was incubated at room temperature for 30 min while being shaken (900 rpm on a tabletop shaker). The resulting lysates were then clarified by centrifugation at 4000 rcf for 10 min at 4 °C, and 100 μL of the clarified lysate was transferred to a well of a SpyCatcher002 coated plate. After loading, the plate was incubated for 90 min at room temperature and 900 rpm using a tabletop shaker to allow for the isopeptide bond to form. The supernatant was discarded, and each well was washed three times with 150 μL of blocking solution.

Alternatively, different amounts of purified tsGGT_xe_ (0.05–100 pmol/well) in lysis buffer^45^ were added to the wells of a SpyCatcher plate instead of lysates from cells and incubated for 90 min at RT. The supernatant was discarded, and each well was washed three times with 150 μL of blocking solution. After that, 100 μL of 1 mM GTA-pNA in 100 mM phosphate buffer at pH 7.4 was added to each well, reaction progress was tracked via release of p-nitroaniline at 37 °C at 405 nm using a microtiter plate reader (Tecan M1000Pro), and concentrations were determined by comparison to a *p*-nitroaniline standard curve. Fitting the Michaelis–Menten equation to the kinetic data was performed with Prism 9 (GraphPad software).

### Activity Comparison of tsGGT_xe__D386X Variants via End Point Measurement of Glutaric Acid

Enzymatic activities of tsGGT_xe__D386X variants obtained from the D386 site saturation library were compared via enzyme immobilization assay combined with end point measurement of released glutaric acid through MS/MS. Therefore, XEc1 cells harboring pDM_tsGGT_xe__D386X variants were grown and tsGGT_xe__D386X variants were immobilized as described in Immobilization of tsGGT_xe_ or Variants Thereof Using SpyCatcher Plates section. In order to compare the hydrolysis activity of immobilized tsGGT_xe__D386X variants, 100 μL of 1 mM GTA-Leu, 200 μM d4-glutaric acid in 10 mM NH_4_HCO_3_ (LC-MS grade, Merck), pH 7.4, was added to each well. The MTP was incubated for 17 h at 37 °C and end point glutaric acid levels were determined via MS/MS using the described glutaric acid quantification method.

### Determination of Immobilized Protein Level via Sandwich-like ELISA

In order to compare the amount of immobilized Spy-tagged proteins, plate wells were rinsed with 1% BSA in PBS and then the plate was incubated with anti-Streptag(II) antibody conjugated with HRP (0.25 μg mL^–1^ in PBS containing 1% BSA) (THE NWSHPQFEK Tag Antibody [HRP], GenScript Biotech, New Jersey) for 2 h at 37 °C. The well was rinsed again with 1% BSA in PBS and 100 μL of colorimetric HRP substrate (TMB, Sigma-Aldrich) was added per well. After 100 s of incubation, the reaction was stopped via addition of 100 μL/well 2 M sulfuric acid. The concentration of the colorimetric product was measured via the absorbance at 450 nm (Tecan M1000Pro).

### Protein Purification and SDS-PAGE Analysis of SpyCatcher002 and tsGGT_xe_ Variants

In order to produce and purify preparative protein amounts, a culture of *E. coli* BL21 DE3 harboring a suitable expression plasmid was grown in 400 mL of LB medium at 37 °C until an OD_600_ of 0.4–0.6 was reached. At this point, protein synthesis was induced by adding IPTG to a final concentration of 0.5 mM and the culture was incubated at 30 °C and 220 rpm. Approximately 17 h later, the cells were harvested by centrifugation at 4000 rcf for 20 min.

In order to obtain preparative amounts of tsGGT_xe_ and variants thereof, we used pDM_tsGGT_xe_ for the expression or variants thereof. The cell pellet was resuspended in 20 mL of 50 mM Tris-HCl, pH 7.4. Cells were then lysed by passing the suspension three times through a homogenizer (EmulsiFlex-C3, Avestin) and the resulting lysate was clarified by centrifugation at 20,000 rcf for 40 min and 4 °C. The supernatant was then filtered through a 0.45 μm syringe filter. The proteins were purified from clarified lysate using the N-terminal Twin-streptag and Strep-Tactin affinity chromatography according to the manufacturer’s protocol (IBA Lifesciences, Göttingen, Germany). Elution fractions were analyzed via SDS-PAGE using Mini-PROTEAN TGX 4–20% precast protein gels (Bio-Rad Laboratories, Hercules).

To obtain preparative amounts of SpyCatcher002, we used plasmid pDEST14-SpyCatcher002 (addgene no. 102827), and cells were resuspended after centrifugation in 20 mM sodium phosphate buffer containing 0.5 M NaCl and 20 mM imidazole at pH 7.4, referred to as loading buffer. Then, the cells were lysed as described above. Clarified lysate was loaded onto a HisTrap HP IMAC column (GE Healthcare, Chicago) by using the KTAprime plus protein purification system. After washing with loading buffer, the His_6_-TEV-SpyCatcher002 protein was eluted by using 20 mM sodium phosphate buffer pH 7.4 containing 0.5 M NaCl and 500 mM imidazole. Elution fractions were analyzed using SDS-PAGE, pooled, and loaded onto a 100 mL Superdex 200 size exclusion column (GE Healthcare), using the same KTAprime plus protein purification system. Size exclusion chromatography was run in PBS, pH = 7.4 with flow set to 1 mL min^–1^. Elution fractions were analyzed via SDS-PAGE using Mini-PROTEAN TGX 4–20% precast protein gels (Bio-Rad Laboratories, Hercules).

### Enzyme Kinetics of tsGGT_xe_ and tsGGT_xe__D386N with GTA-/SBA-Leu

To determine the activity of purified tsGGT_xe_ and tsGGT_xe__D386N with GTA-/SBA-Leu, an enzyme kit (Branched Chain Amino Acid Assay Kit “BCAA”, MAK003, Sigma-Aldrich) was used. The kit couples the formation of the product l-leucine to a colorimetric readout measured at 450 nm. We incubated a fixed amount of purified enzyme (final concentration 8 nM) with different amounts of GTA-Leu or SBA-Leu (final concentrations of 0, 5, 50, 250, 500, 1000, 2500, or 5000 μM) and for each substrate concentration the initial rate of product formation was determined in duplicates. For that, 50 μL of BCAA reaction mixture (46 μL of BCAA assay buffer, 2 μL of BCAA enzyme mix, 2 μL of WST substrate mix) was mixed with 10 μL of GTA-/SBA-Leu solution of a 10-fold concentrated stock in dH_2_O, transferred to a transparent flat-bottom 96-well microtiter plate, and preincubated at 37 °C for 25 min. Meanwhile, an aliquot of 5 μL of purified enzyme (0.01 mg mL^–1^) was mixed with 35 μL of BCAA assay buffer, preheated to 37 °C, and then added to the microtiter plate after preincubation. The plate was then immediately transferred to a microplate reader (Tecan M1000Pro) and the absorbance at 450 nm was measured continuously. The product concentration was determined by comparison to the l-leucine standard curve. Fitting the Michaelis–Menten equation to the kinetic data was performed with Prism 9 (GraphPad software).

## Supporting Information Available

Affinity purification of tsGGT_xe_ (Figure S1); tracking tsGGT_xe_-mediated hydrolysis of GTA-conjugates via quantification of glutaric acid using MS/MS (Figure S2); tracking the reaction progress of tsGGT_xe_ with glutaryl-conjugates by quantifying the release of glutaric acid via MS/MS (Figure S3); cellular localization of tsGGT_xe_ and Δ*S*ignalP-tsGGT_xe_(Figure S4); growth curves showing l-leucine auxotrophic XEc1 under selective and nonselective conditions (Figure S5); DNA sequence analysis of clones obtained during directed evolution and selection with GTA-Leu (Figure S6); purification of His_6_-TEV-SpyCatcher002 via affinity chromatography and SEC followed by analysis via SDS-PAGE (Figure S7); SDS-PAGE analysis of isopeptide formation between purified SpyCatcher002 and tsGGT_xe_ (Figure S8); Michaelis–Menten parameters determined for tsGGT_xe_ and tsGGT_xe__D386N with GTA-Leu and SBA-Leu (Figure S9); DNA oligonucleotides (Table S1); strains (Table S2); plasmids (Table S3); glutaryl-/sulfobutanoyl conjugates (Table S4); supplementary sequences; and supplementary code (PDF)

## References

[ref1] Ignea C.; Raadam M. H.; Koutsaviti A.; Zhao Y.; Duan Y.-T.; Harizani M.; Miettinen K.; Georgantea P.; Rosenfeldt M.; Viejo-Ledesma S. E.; et al. Expanding the terpene biosynthetic code with non-canonical 16 carbon atom building blocks. Nat. Commun. 2022, 13 (1), 5188 10.1038/s41467-022-32921-w. 36057727 PMC9440906

[ref2] Romesberg F. E. Creation, Optimization, and Use of Semi-Synthetic Organisms that Store and Retrieve Increased Genetic Information. J. Mol. Biol. 2022, 434 (8), 167331 10.1016/j.jmb.2021.167331. 34710400

[ref3] Hashimoto K.; Fischer E. C.; Romesberg F. E. Efforts toward Further Integration of an Unnatural Base Pair into the Biology of a Semisynthetic Organism. J. Am. Chem. Soc. 2021, 143 (23), 8603–8607. 10.1021/jacs.1c03860. 34096294 PMC12380253

[ref4] Fischer E. C.; Hashimoto K.; Zhang Y.; Feldman A. W.; Dien V. T.; Karadeema R. J.; Adhikary R.; Ledbetter M. P.; Krishnamurthy R.; Romesberg F. E. New codons for efficient production of unnatural proteins in a semisynthetic organism. Nat. Chem. Biol. 2020, 16 (5), 570–576. 10.1038/s41589-020-0507-z. 32251411 PMC7263176

[ref5] Feldman A. W.; Fischer E. C.; Ledbetter M. P.; Liao J. Y.; Chaput J. C.; Romesberg F. E. A Tool for the Import of Natural and Unnatural Nucleoside Triphosphates into Bacteria. J. Am. Chem. Soc. 2018, 140 (4), 1447–1454. 10.1021/jacs.7b11404. 29338214 PMC5809120

[ref6] Ugwumba I. N.; Ozawa K.; Xu Z.-Q.; Ely F.; Foo J.-L.; Herlt A. J.; Coppin C.; Brown S.; Taylor M. C.; Ollis D. L.; et al. Improving a Natural Enzyme Activity through Incorporation of Unnatural Amino Acids. J. Am. Chem. Soc. 2011, 133 (2), 326–333. 10.1021/ja106416g. 21162578

[ref7] Chen Y.; Jin S.; Zhang M.; Hu Y.; Wu K.-L.; Chung A.; Wang S.; Tian Z.; Wang Y.; Wolynes P. G.; Xiao H. Unleashing the potential of noncanonical amino acid biosynthesis to create cells with precision tyrosine sulfation. Nat. Commun. 2022, 13 (1), 5434 10.1038/s41467-022-33111-4. 36114189 PMC9481576

[ref8] Luo X.; Fu G.; Wang R. E.; Zhu X.; Zambaldo C.; Liu R.; Liu T.; Lyu X.; Du J.; Xuan W.; et al. Genetically encoding phosphotyrosine and its nonhydrolyzable analog in bacteria. Nat. Chem. Biol. 2017, 13 (8), 845–849. 10.1038/nchembio.2405. 28604693 PMC5577365

[ref9] Silhavy T. J.; Kahne D.; Walker S. The bacterial cell envelope. Cold Spring Harb. Perspect. Biol. 2010, 2 (5), a000414 10.1101/cshperspect.a000414. 20452953 PMC2857177

[ref10] Leive L. The barrier function of the gram-negative envelope. Ann. N. Y. Acad. Sci. 1974, 235 (0), 109–129. 10.1111/j.1749-6632.1974.tb43261.x. 4212391

[ref11] Kuenzl T.; Sroka M.; Srivastava P.; Herdewijn P.; Marliere P.; Panke S. Overcoming the membrane barrier: Recruitment of gamma-glutamyl transferase for intracellular release of metabolic cargo from peptide vectors. Metab. Eng. 2017, 39, 60–70. 10.1016/j.ymben.2016.10.016. 27989807

[ref12] Ko W.; Kumar R.; Kim S.; Lee H. S. Construction of Bacterial Cells with an Active Transport System for Unnatural Amino Acids. ACS Synth. Biol. 2019, 8 (5), 1195–1203. 10.1021/acssynbio.9b00076. 30971082

[ref13] Feldman A. W.; Fischer E. C.; Ledbetter M. P.; Liao J.-Y.; Chaput J. C.; Romesberg F. E. A Tool for the Import of Natural and Unnatural Nucleoside Triphosphates into Bacteria. J. Am. Chem. Soc. 2018, 140 (4), 1447–1454. 10.1021/jacs.7b11404. 29338214 PMC5809120

[ref14] Pezo V.; Hassan C.; Louis D.; Sargueil B.; Herdewijn P.; Marlière P. Metabolic Recruitment and Directed Evolution of Nucleoside Triphosphate Uptake in *Escherichia coli*. ACS Synth. Biol. 2018, 7 (6), 1565–1572. 10.1021/acssynbio.8b00048. 29746092

[ref15] Rodríguez-Robles E.; Muller D.; Kunzl T.; Nemat S. J.; Edelmann M. P.; Srivastava P.; Louis D.; Groaz E.; Tiefenbacher K.; Roberts T. M.; et al. Rational design of a bacterial import system for new-to-nature molecules. Metab. Eng. 2024, 85, 26 10.1016/j.ymben.2024.05.005. 38802041

[ref16] Payne J. W.; Smith M. W. Peptide-Transport by Microorganisms. Adv. Microb. Physiol. 1994, 36, 1–80. 10.1016/S0065-2911(08)60176-9. 7942312

[ref17] Payne J. W.; Gilvarg C. Size restriction on peptide utilization in *Escherichia coli*. J. Biol. Chem. 1968, 243 (23), 6291–6299. 10.1016/S0021-9258(18)94490-X. 4881360

[ref18] Chevalier C.; Thiberge J. M.; Ferrero R. L.; Labigne A. Essential role of Helicobacter pylori gamma-glutamyltranspeptidase for the colonization of the gastric mucosa of mice. Mol. Microbiol. 1999, 31 (5), 1359–1372. 10.1046/j.1365-2958.1999.01271.x. 10200957

[ref19] Suzuki H.; Kumagai H.; Tochikura T. gamma-Glutamyltranspeptidase from *Escherichia coli* K-12: formation and localization. J. Bacteriol. 1986, 168 (3), 1332–1335. 10.1128/jb.168.3.1332-1335.1986. 2877975 PMC213642

[ref20] Suzuki H.; Hashimoto W.; Kumagai H. Escherichia-Coli K-12 Can Utilize an Exogenous Gamma-Glutamyl Peptide as an Amino-Acid Source, for Which Gamma-Glutamyl-Transpeptidase Is Essential. J. Bacteriol. 1993, 175 (18), 6038–6040. 10.1128/jb.175.18.6038-6040.1993. 8104180 PMC206686

[ref21] Okada T.; Suzuki H.; Wada K.; Kumagai H.; Fukuyama K. Crystal structures of gamma-glutamyltranspeptidase from *Escherichia coli*, a key enzyme in glutathione metabolism, and its reaction intermediate. Proc. Natl. Acad. Sci. U.S.A. 2006, 103 (17), 6471–6476. 10.1073/pnas.0511020103. 16618936 PMC1458908

[ref22] Suzuki H.; Fukuyama K.; Kumagai H. Bacterial gamma-glutamyltranspeptidases, physiological function, structure, catalytic mechanism and application. Proc. Jpn. Acad. Ser. B Phys. Biol. Sci. 2020, 96 (9), 440–469. 10.2183/pjab.96.033. PMC7725658 33177298

[ref23] Hashimoto W.; Suzuki H.; Yamamoto K.; Kumagai H. Effect of site-directed mutations on processing and activity of gamma-glutamyltranspeptidase of *Escherichia coli* K-12. J. Biochem. 1995, 118 (1), 75–80. 10.1093/oxfordjournals.jbchem.a124894. 8537328

[ref24] Hibi T.; Imaoka M.; Shimizu Y.; Itoh T.; Wakayama M. Crystal structure analysis and enzymatic characterization of gamma-glutamyltranspeptidase from Pseudomonas nitroreducens. Biosci. Biotechnol. Biochem. 2019, 83 (2), 262–269. 10.1080/09168451.2018.1547104. 30507352

[ref25] Sano C.; Itoh T.; Phumsombat P.; Hayashi J.; Wakayama M.; Hibi T. Mutagenesis and structure-based analysis of the role of Tryptophan525 of gamma-glutamyltranspeptidase from Pseudomonas nitroreducens. Biochem. Biophys. Res. Commun. 2021, 534, 286–291. 10.1016/j.bbrc.2020.11.093. 33288198

[ref26] Imaoka M.; Yano S.; Okumura M.; Hibi T.; Wakayama M. Molecular cloning and characterization of gamma-glutamyltranspeptidase from Pseudomonas nitroreducens IFO12694. Biosci. Biotechnol. Biochem. 2010, 74 (9), 1936–1939. 10.1271/bbb.100199. 20834145

[ref27] Suzuki H.; Miwa C.; Ishihara S.; Kumagai H. A single amino acid substitution converts gamma-glutamyltranspeptidase to a class IV cephalosporin acylase (glutaryl-7-aminocephalosporanic acid acylase). Appl. Environ. Microbiol. 2004, 70 (10), 6324–6328. 10.1128/AEM.70.10.6324-6328.2004. 15466585 PMC522061

[ref29] Monneau Y. R.; Ishida Y.; Rossi P.; Saio T.; Tzeng S. R.; Inouye M.; Kalodimos C. G. Exploiting *E. coli* auxotrophs for leucine, valine, and threonine specific methyl labeling of large proteins for NMR applications. J. Biomol. NMR 2016, 65 (2), 99–108. 10.1007/s10858-016-0041-1. 27255761 PMC4936824

[ref30] Sambrook J. Molecular Cloning: A Laboratory Manual, 3rd ed.; Cold Spring Harbor Laboratory Press, 2001; Vol. 1.

[ref31] Adams M. D.; Wagner L. M.; Graddis T. J.; Landick R.; Antonucci T. K.; Gibson A. L.; Oxender D. L. Nucleotide sequence and genetic characterization reveal six essential genes for the LIV-I and LS transport systems of *Escherichia coli*. J. Biol. Chem. 1990, 265 (20), 11436–11443. 10.1016/S0021-9258(19)38417-0. 2195019

[ref32] Komura R.; Aoki W.; Motone K.; Satomura A.; Ueda M. High-throughput evaluation of T7 promoter variants using biased randomization and DNA barcoding. PLoS One 2018, 13 (5), e0196905 10.1371/journal.pone.0196905. 29734387 PMC5937735

[ref33] Conrad T.; Plumbom I.; Alcobendas M.; Vidal R.; Sauer S. Maximizing transcription of nucleic acids with efficient T7 promoters. Commun. Biol. 2020, 3 (1), 439 10.1038/s42003-020-01167-x. 32796901 PMC7429497

[ref34] Ahmadi M. K. B.; Mohammadi S. A.; Makvandi M.; Mamoueie M.; Rahmati M.; Wood D. Column-free purification and coating of SpyCatcher protein on ELISA wells generates universal solid support for capturing of SpyTag-fusion protein from the non-purified condition. Protein Expr. Purif. 2020, 174, 105650 10.1016/j.pep.2020.105650. 32360597 PMC7189850

[ref35] Studier F. W. Protein production by auto-induction in high density shaking cultures. Protein Expr. Purif. 2005, 41 (1), 207–234. 10.1016/j.pep.2005.01.016. 15915565

[ref36] Imaoka M.; Yano S.; Okumura M.; Hibi T.; Wakayama M. Molecular Cloning and Characterization of γ-Glutamyltranspeptidase fromPseudomonas nitroreducensIFO12694. Biosci. Biotechnol. Biochem. 2010, 74 (9), 1936–1939. 10.1271/bbb.100199. 20834145

[ref37] Pestalozzi L. M. Directed Evolution of Tobacco Etch Virus Protease Towards Higher In Vitro Activity, Ph.D. Thesis; ETH Zurich: Zurich, 2019.

[ref38] Gibson D. G.; Young L.; Chuang R. Y.; Venter J. C.; Hutchison C. A. 3rd; Smith H. O. Enzymatic assembly of DNA molecules up to several hundred kilobases. Nat. Methods 2009, 6 (5), 343–345. 10.1038/nmeth.1318. 19363495

[ref39] Cline J.; Braman J. C.; Hogrefe H. H. PCR fidelity of pfu DNA polymerase and other thermostable DNA polymerases. Nucleic Acids Res. 1996, 24 (18), 3546–3551. 10.1093/nar/24.18.3546. 8836181 PMC146123

[ref40] Datsenko K. A.; Wanner B. L. One-step inactivation of chromosomal genes in *Escherichia coli* K-12 using PCR products. Proc. Natl. Acad. Sci. U.S.A. 2000, 97 (12), 6640–6645. 10.1073/pnas.120163297. 10829079 PMC18686

[ref41] Panke S.; Meyer A.; Huber C. M.; Witholt B.; Wubbolts M. G. An alkane-responsive expression system for the production of fine chemicals. Appl. Environ. Microbiol. 1999, 65 (6), 2324–2332. 10.1128/AEM.65.6.2324-2332.1999. 10347009 PMC91344

[ref42] Adusumilli R.; Mallick P. Data Conversion with ProteoWizard msConvert. Methods Mol. Biol. 2017, 1550, 339–368. 10.1007/978-1-4939-6747-6_23. 28188540

[ref43] Keeble A. H.; Banerjee A.; Ferla M. P.; Reddington S. C.; Anuar I.; Howarth M. Evolving Accelerated Amidation by SpyTag/SpyCatcher to Analyze Membrane Dynamics. Angew. Chem., Int. Ed. 2017, 56 (52), 16521–16525. 10.1002/anie.201707623. PMC5814910 29024296

[ref44] Zakeri B.; Fierer J. O.; Celik E.; Chittock E. C.; Schwarz-Linek U.; Moy V. T.; Howarth M. Peptide tag forming a rapid covalent bond to a protein, through engineering a bacterial adhesin. Proc. Natl. Acad. Sci. U.S.A. 2012, 109 (12), E690–697. 10.1073/pnas.1115485109. 22366317 PMC3311370

[ref45] Glauche F.; Pilarek M.; Bournazou M. N. C.; Grunzel P.; Neubauer P. Design of experiments-based high-throughput strategy for development and optimization of efficient cell disruption protocols. Eng. Life Sci. 2017, 17 (11), 1166–1172. 10.1002/elsc.201600030. 32624744 PMC6999527

