# Supplementary material for: Characterizing and Tailoring the Substrate Profile of a γ-Glutamyltransferase Variant
Source: ACS Synth Biol. 2024 Aug 12;13(9):2969–81. doi: 10.1021/acssynbio.4c00364 (PMC11421214; doi:10.1021/acssynbio.4c00364)
Supplement: Supplementary file 1 — sb4c00364_si_001.pdf [file sb4c00364_si_001.pdf]

Characterizing and tailoring the substrate profile of a gamma-glutamyltransferase variant

David Mueller[a], *Remo Baettig*[a], *Tilmann Kuenzl*[a], *Emilio Rodríguez-Robles*[a], *Tania Michelle Roberts*[a], *Philippe Marlière*[b] *& Sven Panke**[a]

[a] Department of Biosystems Science and Engineering, ETH Zürich, 4056 Basel, Switzerland

[b] TESSSI, The European Syndicate of Synthetic Scientists and Industrialists, 75002 Paris, France

*sven.panke@bsse.ethz.ch

Supplementary Figures

Supplementary Figure S1

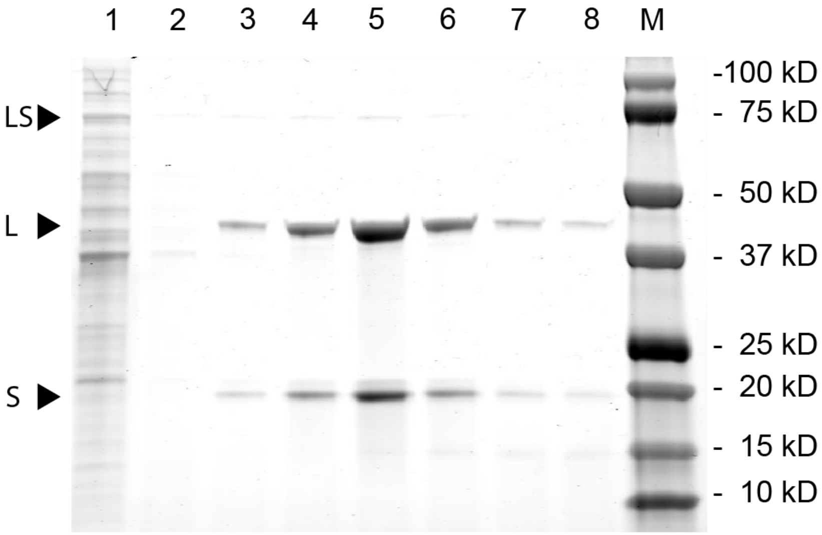

Supplementary Figure S1: Affinity purification of tsGGT$_{xe}$: SDS PAGE of different steps of the affinity purification process using an N-terminal Twin-streptag and a Strep-tactin affinity matrix. tsGGT$_{xe}$ was produced in strain *E. coli* BL21 DE3 [pDM_tsGGT$_{xe}$] and isolated from lysate using the N-terminal Twin-streptag and a Strep-tactin (IBA Lifesciences) gravity flow column. Fractions obtained during this process were analyzed via SDS-PAGE. Lane 1: cell lysate; lane 2: wash; lanes 3-8: elution; M: molecular size marker. L = large tsGGT$_{xe}$ subunit (exp. 42.4 kDa), S = small tsGGT$_{xe}$ subunit (exp. 20.8 kDa) and LS = complex of large & small tsGGT$_{xe}$ subunit (exp. 63.2 kDa). M = Precision Plus Protein Dual color Plus (Bio-Rad).

Supplementary Figure S2

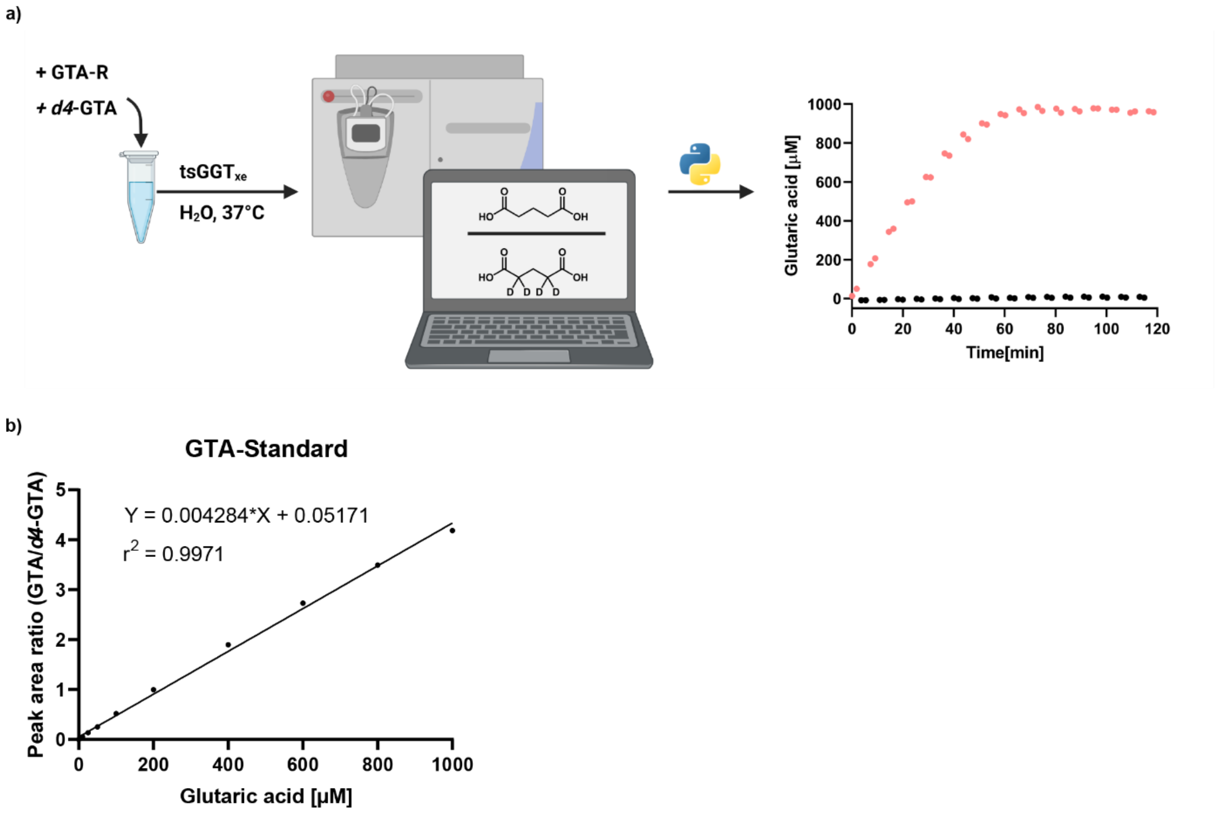

Supplementary Figure S2: Tracking $tsGGT_{xe}$-mediated hydrolysis of GTA-conjugates via quantification of glutaric acid using MS/MS. a) Schematic illustration of the MS/MS based enzyme activity screen used to determine the substrate promiscuity of $tsGGT_{xe}$. A fixed amount of purified $tsGGT_{xe}$ was incubated with a member of the GTA-conjugate library and a known amount d4-glutaric acid in MS compatible buffer at 37°C in the autosampler of an HPLC device. The reaction mixture was injected several times into the MS/MS and released glutaric acid as well as d4-glutaric acid were quantified. A python script was used to integrate the area under the peaks, calculating the analyte to internal standard ratio and to later determine initial velocity of reaction progress. b) Standard curve used for quantification of free glutaric acid in samples.

Supplementary Figure S3

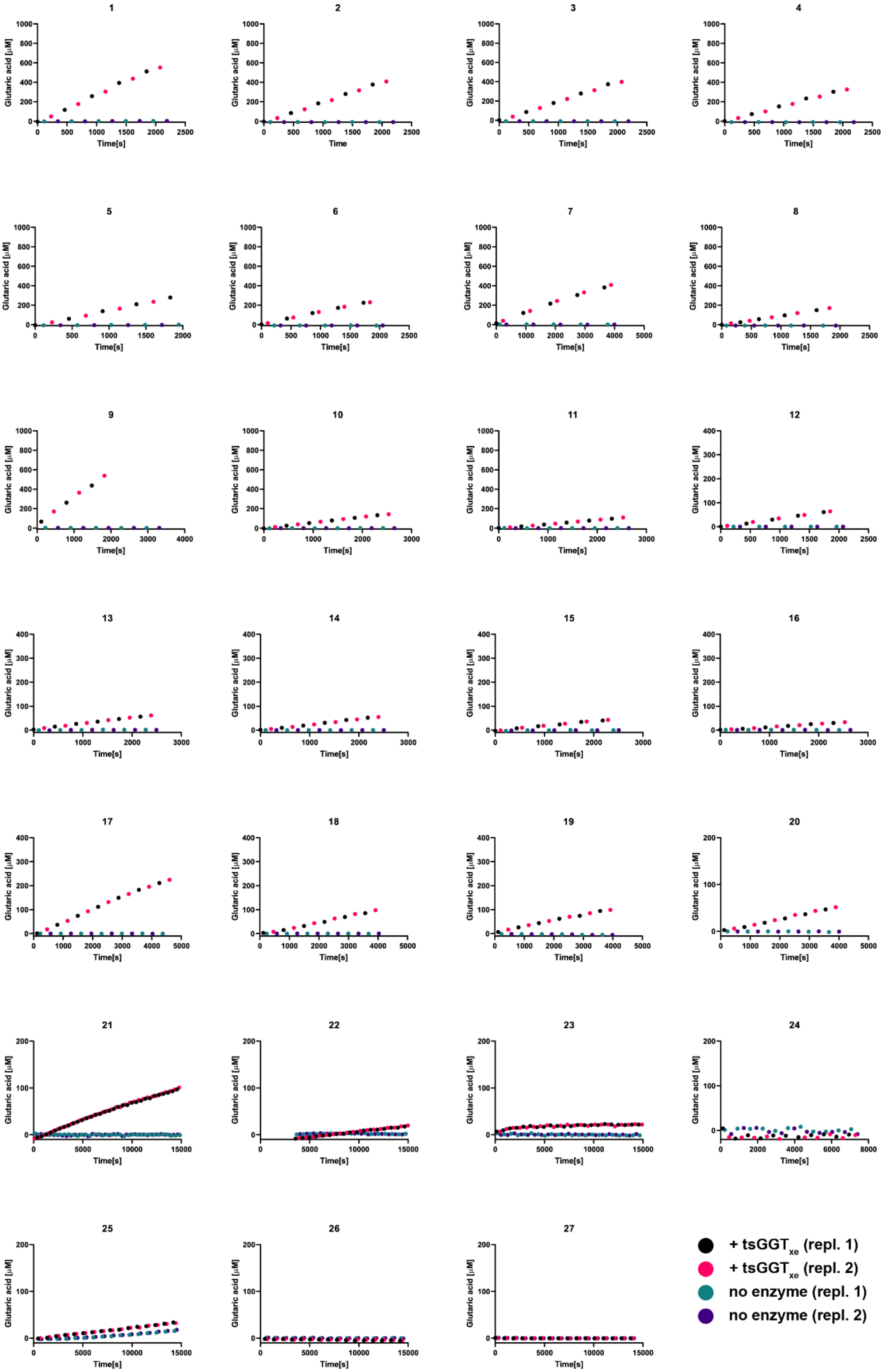

Supplementary Figure S3: Tracking the reaction progress of $tsGGT_{xe}$ with glutaryl-conjugates by quantifying the release of glutaric acid via MS/MS. Initial velocities of reaction progress curves were calculated by individually incubating 1 mM of each GTA-conjugate together with 200 µM d4-glutaric acid and a fixed amount of purified $tsGGT_{xe}$ (27nM enzyme for compounds **1**-**8** and **10**-**16**. Because of low activities, four times more enzyme (108 nM) was used for compounds **9** and **17**-**27**). As a control, substrate solutions where no enzyme was added were measured to track $tsGGT_{xe}$ independent hydrolysis, this effect was most prevalent with GTA-Indole (**25**).

Supplementary Figure S4

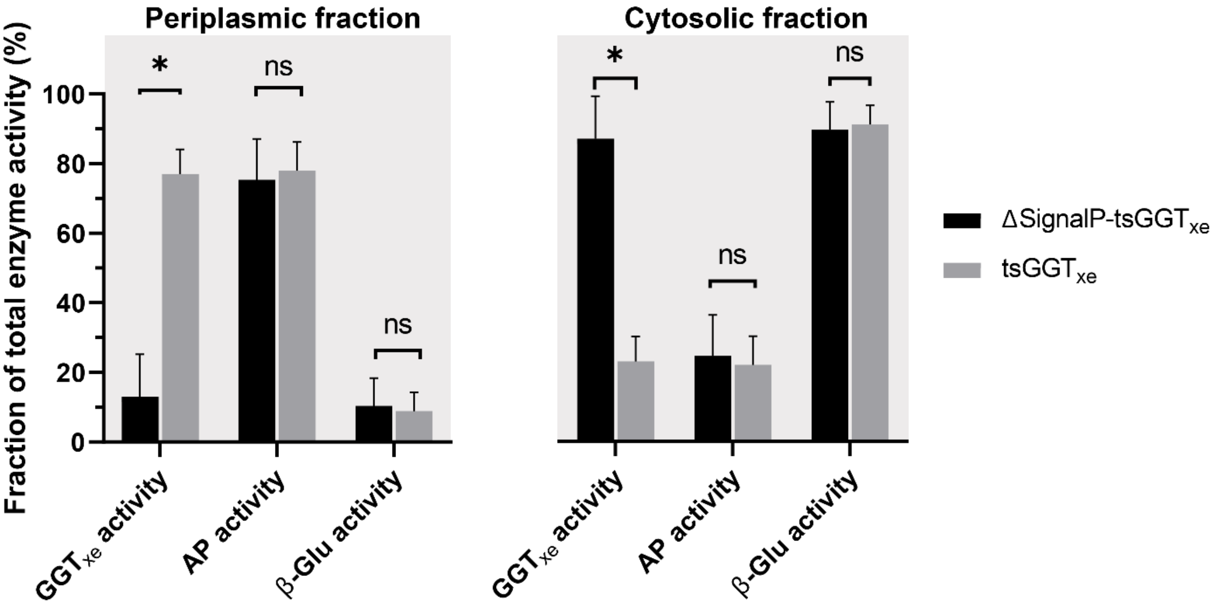

Supplementary Figure S4: Cellular localization of $tsGGT_{xe}$ and ΔSignalP-$tsGGT_{xe}$ Periplasmic and cytosolic fractions of XEc1 expressing either $tsGGT_{xe}$ or ΔSignalP-$tsGGT_{xe}$ were separated, and fractions were analyzed for the presence of GGT_{xe} hydrolysis activity, using GTA-pNA as substrate. AP = Alkaline phosphatase activity was used as a periplasmic marker and β-Glu (β-Glucuronidase) activity served as a cytosolic marker. An unpaired t-test was used to compare the mean of enzyme activity (ns, not significant (p-value ≥ 0.05); *, significant (p-value < 0.05)).

Supplementary Figure S5

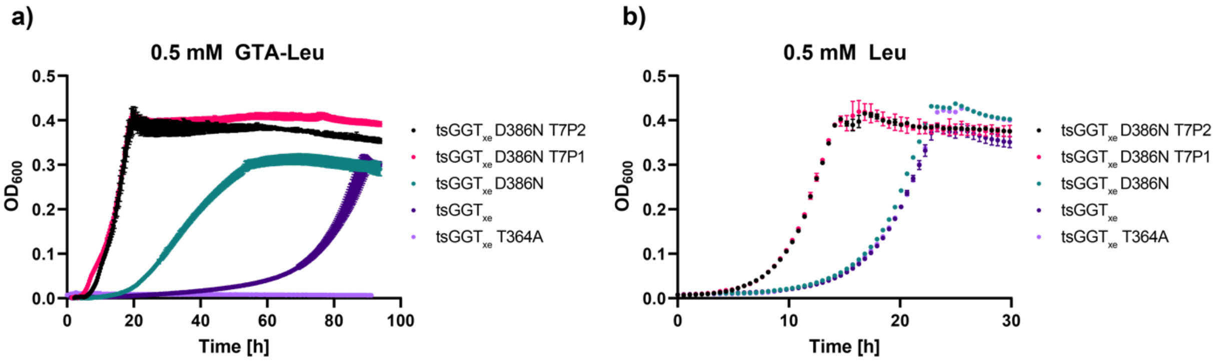

Supplementary Figure S5: Growth curves showing L-leucine auxotrophic XEc1 under selective and non-selective conditions. a) Selective conditions: XEc1[pDM_tsGGT$_{xe}$] cells producing tsGGT$_{xe}$, XEc1[pDM_tsGGT$_{xe}$_T364A] producing catalytically inactive tsGGT$_{xe}$_T364A, or XEc1[pDM_tsGGT$_{xe}$_X] expressing the gene of the D386N variant of tsGGT$_{xe}$ with or without changed expression signals were grown at 37°C in M9 minimal medium supplemented with 0.4 % fructose, 50 µM IPTG, 50 µg mL^{-1} kanamycin and 0.5 mM GTA-Leu in a 96-well microtiter plate. Growth was determined by tracking OD$_{600}$ over time. b) Non-selective conditions: XEc1[pDM_tsGGT$_{xe}$] cells producing tsGGT$_{xe}$, XEc1[pDM_tsGGT$_{xe}$_T364A] producing catalytically inactive tsGGT$_{xe}$_T364A, or XEc1[pDM_tsGGT$_{xe}$_X] expressing the gene of the D386N variant of tsGGT$_{xe}$ with or without changed expression signals were grown at 37°C in M9 minimal medium supplemented with 0.4 % fructose, 50 µM IPTG, 50 µg mL^{-1} kanamycin and 0.5 mM L-leucine.

Supplementary Figure S6

a)

Clone	Mutations in gene
1	D386N
2	D386N
3	N379N, D386N, G390G
4	D386N
5	D386N, H489H
6	D386N
7	D386N
8	D386N

b)

Clone	Mutations in gene	Mutations in promoter region
1	D386N, A424A, L468L, L476L	TAATCGGCTCACTATAGG
2	D386N, A424A, L468L, L476L	TAATCGGCTCACTATAGG
3	D386N, A424A, L468L, L476L	TAATCGGCTCACTATAGG
4	D386N	TAATCGACTCACTATAGT
5	D386N, L476L	TAATCGACTCACTATAGT

c)

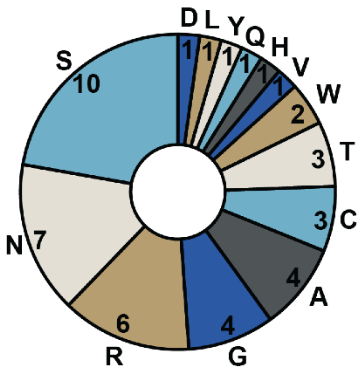

Supplementary Figure S6: DNA sequence analysis of clones obtained during directed evolution and selection with GTA-Leu. a) Plasmids of all eight clones showing increased growth rate on GTA-Leu after the first round of selection were extracted and the small subunit was sequenced. All eight clones showed a common mutation at position 386 which changed the corresponding amino acid from aspartate to asparagine. b) After the second round of selection, plasmids of clones with increased growth rate compared to the parent ($tsGGT_{xe}$_D386N), were sequenced. In addition to the sequencing of the small subunit, the sequence of the promoter and the large subunit were determined as well. Only silent mutations were observed in the coding sequence, but all five clones showed either changes in the T7 promoter sequence or in bases close to the transcriptional start site. c) Pie plot showing the frequency of different amino acids at residue 386 that allowed for increased growth under selective conditions. A total of n = 45 clones from the NNK site saturation library of residue 386 were sequenced.

Supplementary Figure S7

a)

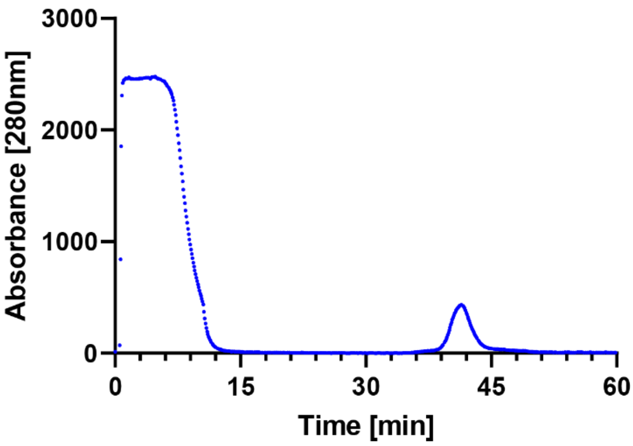

b)

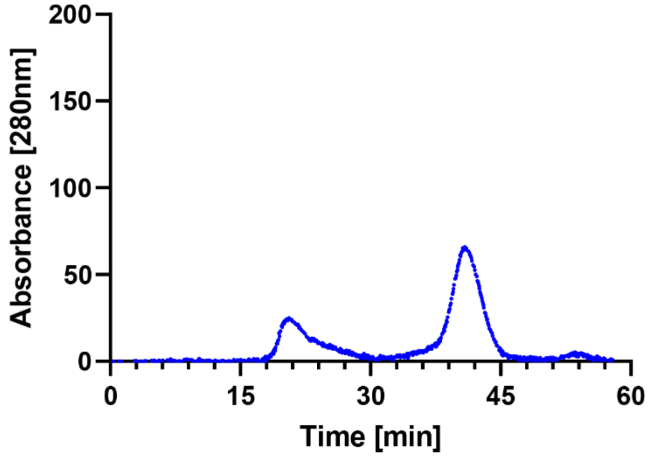

c)

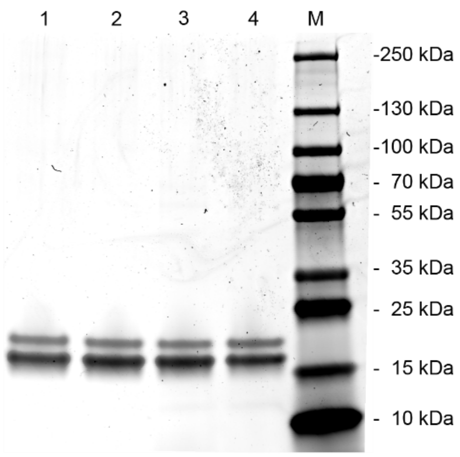

Supplementary Figure S7: Purification of His$_6$-TEV-SpyCatcher002 via affinity chromatography and SEC followed by analysis via SDS-PAGE. His$_6$-TEV-SpyCatcher002 variant was expressed in BL21 DE3 cells using the pDEST14-SpyCatcher002 expression vector (addgene #194557). Lysate was prepared and SpyCatcher002 purified using a NI-NTA affinity column a) Chromatogram showing the absorbance at 280 nm during the NI-NTA affinity chromatography. His$_6$-TEV-SpyCatcher002 protein referred to as SpyCatcher002 was eluted by gradually increasing the imidazole concentration from 20 to 500 mM. The eluted peak between 35-45 min was collected and further purified using a sephadex 200 size exclusion column. b) Size exclusion chromatography of combined elution fractions obtained after NI-NTA purification. The separation was performed using PBS at pH 7.4. The peak at 40min, corresponding to an approximate molecular weight of 16 kDa (expected: 15.8 kDa) was collected and fractions analyzed via SDS-PAGE. c) SDS-page confirmed the purity of SpyCatcher002, lane 1-4 contain the SpyCatcher002 elution fraction after size exclusion chromatography. The second band is most likely a not fully denatured SpyCatcher002 protein. M = PageRuler Plus (ThermoFisher Scientific)

Supplementary Figure S8

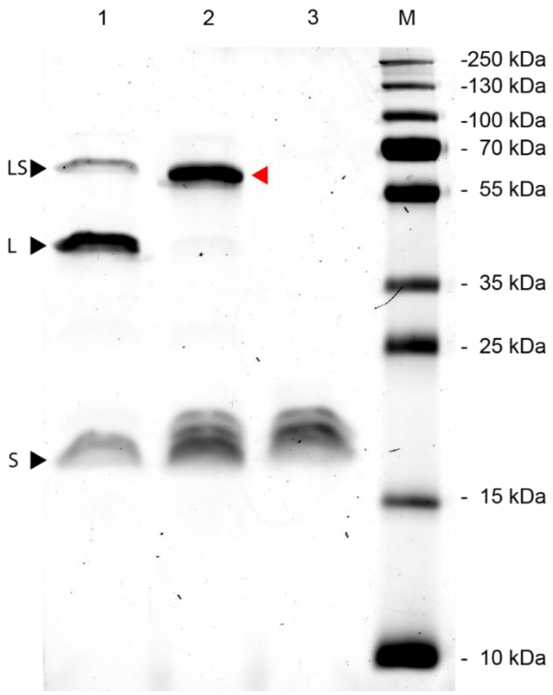

Supplementary Figure S8: SDS-PAGE analysis of isopeptide formation between purified SpyCatcher002 and tsGGT$_{xe}$. In order to determine if the SpyTag002, located between the N-terminal Twin-streptag and the coding sequence of tsGGT$_{xe}$, is accessible and therefore able to form a covalent isopeptide bond with SpyCatcher002, a fixed amount of purified tsGGT$_{xe}$ and purified SpyCatcher002 (1µg each, corresponding to a 4-fold excess of SpyCatcher002 to tsGGT$_{xe}$) were mixed and incubated in PBS at room temperature for 90 min. As a control, 1µg of SpyCatcher002 as well as 1µg of tsGGT$_{xe}$ were incubated separately, all three samples were then analyzed via SDS-PAGE. Lane 1: tsGGT$_{xe}$, lane 2: SpyCatcher002 and tsGGT$_{xe}$ mix, lane 3: SpyCatcher002. Red triangle shows the fusion protein of SpyCatcher002 with the large subunit of tsGGT$_{xe}$ (expected: 58.1 kDa). L = large tsGGT$_{xe}$ subunit (expected: 42.4 kDa), S = small tsGGT$_{xe}$ subunit (expected: 20.8 kDa) and LS = complex of large & small tsGGT$_{xe}$ subunit (expected: 63.2 kDa), SpyCatcher002 (expected: 15.7 kDa). M = PageRuler Plus (ThermoFisher Scientific)

Supplementary Figure S9

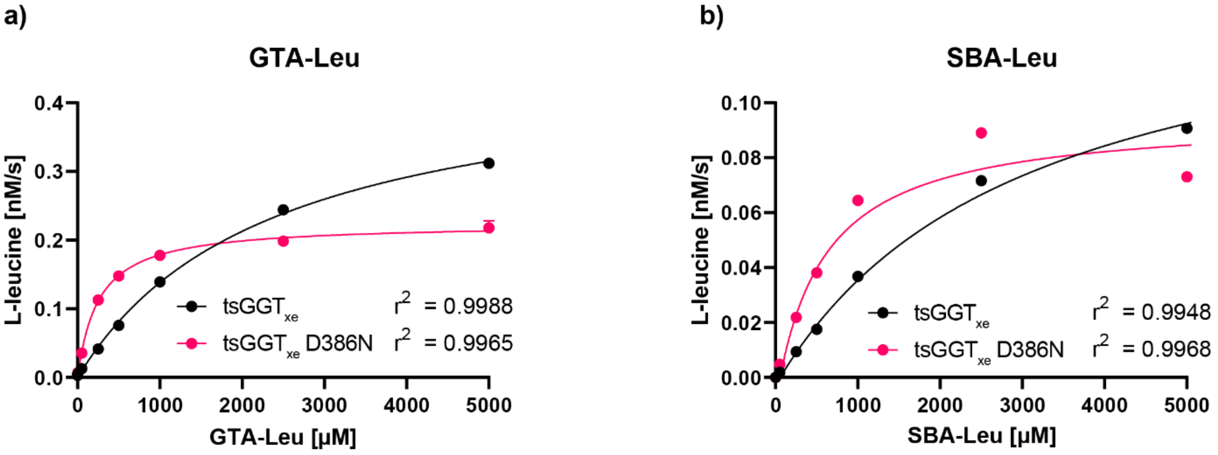

Supplementary Figure S9: Michaelis Menten parameters determined for tsGGT$_{xe}$ and tsGGT$_{xe}$_D386N with GTA-Leu and SBA-Leu. In order to determine kinetic parameters for tsGGT$_{xe}$ and tsGGT$_{xe}$_D386N using a) GTA-Leu and b) SBA-Leu as substrates, we used a colorimetric enzyme activity assay based on a commercial branched-chain amino acid quantification kit, that uses L-leucine as a substrate and forms a colorimetric product with increased absorbance at 450 nm. A fixed concentration of purified tsGGT$_{xe}$ and tsGGT$_{xe}$_D386N (8 nM) was incubated with different concentrations of GTA- or SBA-Leu and initial velocities were calculated. Fitting the Michaelis-Menten equation to the kinetic data was performed with Prism 9 (GraphPad software).

Supplementary Data

Supplementary Table S1: DNA oligonucleotides

**Primer**	**Nucleotide Sequence 5’-3’**
oDAM102	CGAGAAAGTCCGCCCGG
oDAM103	CGTGGTCTGGTGCGGCT
oDAM104	CGGCGTCGGGATGGTGG
oDAM105	GCGCGAATTCTCAGGGTTTG
oDAM033	CAGCAACACCTACACCCTCAACTGGNNKTTCGGCAGCGGCGTGGTGGTC
oDAM034	CAGTTGAGGGTGTAGGTGTTGCTG
oDAM001	GGAATTGTTATCCGCTCACAATTC
oDAM002	GAATTCGCGCGGCCGCG
oDAM018	GTGCCTACTATCGTGATGGTG
oDAM055	GGTTATCCAGGCTAAAATCG
oDAM056	CGATTTTAGCCTGGATAACC
ERR057F	TTATATACCAGGCTTAGCTGGGGTTGCCCCTTAATCTCTGGAGAATAACGATTAATTCCGGGGATCCGTC
ERR057R	CGGCTTGCCCTGACAAAATAGCCCTCTTCCCACGAAGAGGGCCGCTAACCTGGAGCTGCTTCGAAGTTC
ERR058F	GCTCAATGTATCACACAG
ERR058R	GCTGATTCAACGAACTATC

Supplementary Table S2: Strains

**Strain**	**Relevant characteristics**	**Source**
E. coli BL21 DE3	F⁻ ompT hsdS$_B$ (r$_B$⁻ m$_B$⁻) gal dcm (DE3)	Novagen
E. coli “E. cloni 10G Elite”	F- mcrA Δ(mrr-hsdRMS-mcrBC) endA1 recA1 Φ80dlacZΔM15 ΔlacX74 araD139 Δ(ara,leu)7697 galU galK rpsL nupG λ- tonA	Lucigen
E. coli “SIG10 Ultra”	F- mcrA Δ(mrr-hsdRMS-mcrBC) endA1 recA1 Φ80dlacZΔM15 ΔlacX74 araD139 Δ(ara,leu)7697galU galK rpsL nupG λ- tonA (StrR)	Sigma-Aldrich
E. coli MG1655 DE3 Δ*leuABCD* Δ*ggt* «XEc1»	K-12 DE3 F– λ– *ilvG*– *rfb*-50 *rph*-1 Δ*leuABCD* Δ*ggt*	This study

Supplementary Table S3: Plasmids

**Plasmid**	**Relevant characteristics**	**Source**
pDEST14-SpyCatcher002	pDEST14, Resistance: Ampicillin, ORI:pBR322, Expression casette: P_{T7}, His$_6$-TEV-cleavage-site-SpyCatcher002	Addgene[43] #102827
pGFP	pSEVA261 based[46], Resistance: Kanamycin, ORI:p15a,	Luzius Pestalozzi[37]

	Expression casette: *lac*I-P_{T7}-*lac*O,	
pDM_ΔSignalP-tsGGT$_{xe}$	pGFP based<br><br>Resistance: Kanamycin,<br><br>ORI:p15a,<br><br>Expression casette: *lac*I-P_{T7}-*lac*O,<br><br>tsGGT$_{xe:}$ Twin-streptag-SpyTag-GSGESGELT-linker-Δ24N-*pn*GGT	This study
pDM_tsGGT$_{xe}$	pGFP based<br><br>Resistance: Kanamycin,<br><br>ORI:p15a,<br><br>Expression casette: *lac*I-P_{T7}-*lac*O,<br><br>SP + tsGGT$_{xe:}$ SignalPeptide (N24) of *pn*GGT fused to Twin-streptag-SpyTag-GSGESGELT-linker-Δ24N-*pn*GGT	This study
pDM_tsGGT$_{xe}$_D386N	pGFP based<br><br>Resistance: Kanamycin,<br><br>ORI:p15a,<br><br>Expression casette: *lac*I-P_{T7}-*lac*O,<br><br>SP + tsGGT$_{xe:}$ SignalPeptide (N24) of *pn*GGT fused to Twin-streptag-SpyTag-GSGESGELT-linker-Δ24N-*pn*GGT_D386N	This study
pDM_tsGGT$_{xe}$_T364A	pGFP based<br><br>Resistance: Kanamycin,<br><br>ORI:p15a,<br><br>Expression casette: *lac*I-P_{T7}-*lac*O,	This study

	SP + tsGGT$_{xe:}$ SignalPeptide (N24) of *pn*GGT fused to Twin-streptag-SpyTag-GSGESGELT-linker-Δ24N-*pn*GGT_T364A	

Supplementary Table S4: Glutaryl-/Sulfobutanoyl conjugates

Sulfobutanoyl-conjugates

**Compound**	**Name**	**CAS number**	**Purity [%]**	**Source**
SBA-Leucine	Sulfobutanoyl-L-leucine	N/A	98	Pepscan

Glutaryl-conjugates

**Compound Number**	**Name**	**CAS number / Chemspace ID**	**Purity [%]**	**Source**
1	4-[(4-nitrophenyl)carbamoyl]butanoic acid	5502-64-7	95	Chemspace
2	sodium 4-[(2-phenylethyl)carbamoyl]butanoate	2416230-61-8	95	Chemspace
3	4-[(naphthalen-1-yl)carbamoyl]butanoic acid	296275-32-6	≥ 95	Life Chemicals Inc.
4	(2S)-6-amino-2-(4-carboxybutanamido)hexanoic acid	76477-39-9	95	Chemspace
5	Glutaryl-L-phenylalanin-4-nitroanilid	5800-34-0	≥ 98	Sigma-Aldrich
6	4-[(2-methylphenyl)carbamoyl]butanoic acid	78648-34-7	95	Chemspace
7	4-carbamoylbutanoic acid	25335-74-4	95	Chemspace

8	sodium 4-{[4-(ethoxycarbonyl)phenyl]carbamoyl}butanoate	147891-15-4	95	Chemspace
9	4-[(2-methoxy-2-oxoethyl)carbamoyl]butanoic acid	587022-78-4	95	Chemspace
10	O-(N-Biotinyl-3-aminopropyl)-O'-(N-glutaryl-3-aminopropyl)-diethyleneglycol · DIPEA	1205744-09-7	≥ 98	Novabiochem
11	4-(tert-butylcarbamoyl)butanoic acid	540795-50-4	95	Chemspace
12	sodium 4-[(adamantan-1-yl)carbamoyl]butanoate	2416236-33-2	95	Chemspace
13	Glutaryl-glycyl-L-arginine 7-amido-4-methylcoumarin hydrochloride	103213-40-7	> 97	Iris Biotech
14	4-[(propan-2-yl)carbamoyl]butanoic acid	59481-43-5	98	Chemspace
15	sodium 5-(carbamoylamino)-5-oxopentanoate	2416235-21-5	95	Chemspace
16	4-({2-[2-(2-hydroxyethoxy)ethoxy]ethyl}carbamoyl)butanoic acid	CSSB00059238957	95	Chemspace
17	4-{[(1S)-1-carboxy-2-methylpropyl]carbamoyl}butanoic acid	85562-63-6	≥ 95	Chemspace
18	(2S,3S)-2-(4-carboxybutanamido)-3-methylpentanoic acid	2624109-55-1	95	Chemspace

19	4-{[(1S)-1-carboxy-2-(4-hydroxy-3-nitrophenyl)ethyl]carbamoyl}butanoic acid	CSSB06657271944	95	Chemspace
20	4-{[(1S)-1-carboxy-2-phenylethyl]carbamoyl}butanoic acid	37466-27-6	95	Chemspace
21	(2S)-2-(4-carboxybutanamido)-4-methylpentanoic acid	124397-74-6	95	Chemspace
22	4-{[(1S)-2-carbamoyl-1-carboxyethyl]carbamoyl}butanoic acid	CSMB06633714733	95	Chemspace
23	4-[(carboxymethyl)carbamoyl]butanoic acid	17686-38-3	95	Chemspace
24	(2S)-2-(4-carboxybutanamido)butanedioic acid	1373771-20-0	95	Chemspace
25	5-(1H-indol-1-yl)-5-oxopentanoic acid	123154-20-1	95	Chemspace
26	4-(dimethylcarbamoyl)butanoic acid	151675-59-1	95	Chemspace
27	4-[(2,6-dimethylphenyl)carbamoyl]butanoic acid	201137-73-7	95	Chemspace
	Glutaric acid (2,2,4,4-D4)	19136-99-3	98	CIL Inc.

Supplementary Sequences

pDM_ΔSignalP-tsGGT$_{xe}$

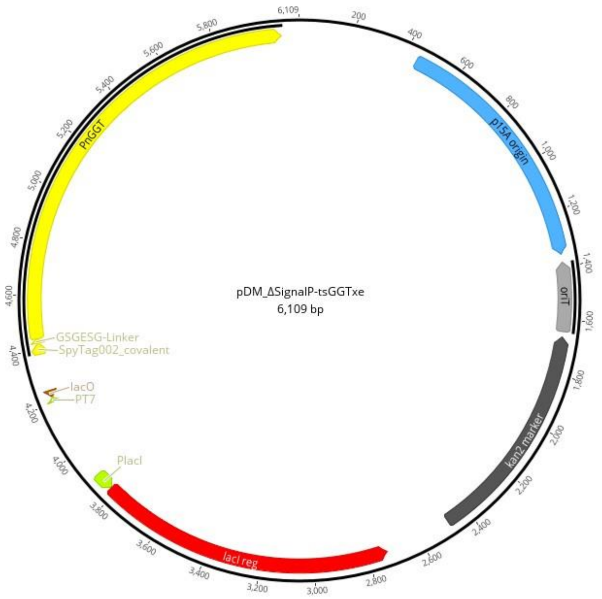

AGGCATCAAATAAAACGAAAGGCTCAGTCGAAAGACTGGGCCTTTCGTTTTATCTGTTGTTTGTCGGTGAACGCTCTCCT
GAGTAGGACAAATCCGCCGCCCTAGACAGCTGGGCGCGCCAAATACGCCCGGTAGTGATCTTATTTCATTATGGTGAAAG
TTGGAACCTCTTACGTGCCGATCAACGTCTCATTTTCGCCAAAAGTTGGCCCAGGGCTTCCCGGTATCAACAGGGACACC
AGGATTTATTTATTCTGCGAAGTGATCTTCCGTCACAGGTATTTATTCGGCGCAAAGTGCGTCGGGTGATGCTGCCAACT
TACTGATTTAGTGTATGATGGTGTTTTTGAGGTGCTCCAGTGGCTTCTGTTTCTATCAGCTGTCCCTCCTGTTCAGCTAC
TGACGGGGTGGTGCGTAACGGCAAAAGCACCGCCGGACATCAGCGCTAGCGGAGTGTATACTGGCTTACTATGTTGGCAC
TGATGAGGGTGTCAGTGAAGTGCTTCATGTGGCAGGAGAAAAAAGGCTGCACCGGTGCGTCAGCAGAATATGTGATACAG
GATATATTCCGCTTCCTCGCTCACTGACTCGCTACGCTCGGTCGTTCGACTGCGGCGAGCGGAAATGGCTTACGAACGGG
GCGGAGATTTCCTGGAAGATGCCAGGAAGATACTTAACAGGGAAGTGAGAGGGCCGCGGCAAAGCCGTTTTTCCATAGGC
TCCGCCCCCCTGACAAGCATCACGAAATCTGACGCTCAAATCAGTGGTGGCGAAACCCGACAGGACTATAAAGATACCAG
GCGTTTCCCCCTGGCGGCTCCCTCGTGCGCTCTCCTGTTCCTGCCTTTCGGTTTACCGGTGTCATTCCGCTGTTATGGCC
GCGTTTGTCTCATTCCACGCCTGACACTCAGTTCCGGGTAGGCAGTTCGCTCCAAGCTGGACTGTATGCACGAACCCCCC
GTTCAGTCCGACCGCTGCGCCTTATCCGGTAACTATCGTCTTGAGTCCAACCCGGAAAGACATGCAAAAGCACCACTGGC
AGCAGCCACTGGTAATTGATTTAGAGGAGTTAGTCTTGAAGTCATGCGCCGGTTAAGGCTAAACTGAAAGGACAAGTTTT
GGTGACTGCGCTCCTCCAAGCCAGTTACCTCGGTTCAAAGAGTTGGTAGCTCAGAGAACCTTCGAAAAACCGCCCTGCAA
GGCGGTTTTTTCGTTTTCAGAGCAAGAGATTACGCGCAGACCAAAACGATCTCAAGAAGATCATCTTATTAATCAGATAA
AATATTTCTAGCATGAGCGGATACATATTTGAATGTATTTAGAAAAATAAACAAATAGGGGTTCCGCGCACATTTCCCCG
AAAAGTGCCACCTGCTAGGGCCGGCCTACGGCCAGCCTCGCAGAGCAGGATTCCCGTTGAGCACCGCCAGGTGCGAATAA
GGGACAGTGAAGAAGGAACACCCGCTCGCGGGTGGGCCTACTTCACCTATCCTGCCCGGCTGACGCCGTTGGATACACCA
AGGAAAGTCTACACGAACCCTTTGGCAAAATCCTGTATATCGTGCGAAAAAGGATGGATATACCGAAAAAATCGCTATAA
TGACCCCGAAGCAGGGTTATGCAGCGGAAAAGGACAACGCGCGGACCGCGGTCCAATTAATTATTAGAAAAATTCATCCA
GCATCAGATGAAATTGCAGTTTGTTCATATCCGGATTATCAATGCCATATTTCTGAAACAGACGTTTTTGCAGGCTCGGG
CTAAATTCGCCCAGGCAGTTCCACAGAATGGCCAGATCCTGATAACGATCCGCAATGCCCACACGGCCCACATCAATGCA
GCCAATCAGTTTGCCTTCATCGAAAATCAGGTTATCCAGGCTAAAATCGCCGTGGGTCACCACGCTATCCGGGCTAAACG
GCAGCAGTTTATGCATTTCTTTCCACACCTGTTCCACCGGCCAGCCGTTACGTTCATCATCAAAATCGCTCGCATCCACC
AGGCCGTTGTTCATACGGCTCTGCGCCTGGGCCAGACGAAACACACGATCGCTGTTAAACGGGCAGTTGCACACCGGAAT
GCTATGCAGACGACGCAGAAACACGGCCAGCGCATCCACAATGTTTTCGCCGCTATCCGGATATTCTTCCAGCACCTGAA
ACGCGGTTTTGCCCGGAATCGCGGTGGTCAGCAGCCACGCATCATCCGGGGTGCGAATAAAATGTTTAATGGTCGGCAGC
GGCATAAATTCGGTCAGCCAGTTCAGACGCACCATTTCATCGGTCACATCGTTCGCCACGCTGCCTTTGCCATGTTTCAG
AAACAGTTCCGGCGCATCCGGTTTGCCATACAGACGATAAATGGTCGCGCCGCTCTGACCCACGTTATCACGCGCCCATT
TATAGCCATACAGATCCGCATCCATGTTGCTGTTCAGACGCGGACGGCTACAGCTCGTTTCACGCTGAATATGGCTCATA

ACACCCCTTGTATTACTGTTTATGTAAGCAGACAGTTTTATTGTTCATGATGATATATTTTTATCTTGTGCAATGTAACA
TCAGAGATTTTGAGACACAAATTTAAATCGTAATTATTGGGGACCCCTGGATTCTCACCAATAAAAAACGCCCGGCGGCA
ACCGAGCGTTCTGAACAAATCCAGATGGAGTTCTGAGGTCATTACTGGATCTATCAACAGGAGTCCAAGACTAGTTTAAT
TGCGTTGCGCTCACTGCCCGCTTTCCAGTCGGGAAACCTGTCGTGCCAGCTGCATTAATGAATCGGCCAACGCGCGGGGA
GAGGCGGTTTGCGTATTGGGCGCCAGGGTGGTTTTTCTTTTCACCAGTGAGACGGGCAACAGCTGATTGCCCTTCACCGC
CTGGCCCTGAGAGAGTTGCAGCAAGCGGTCCACGCTGGTTTGCCCCAGCAGGCGAAAATCCTGTTTGATGGTGGTTAACG
GCGGGATATAACATGAGCTGTCTTCGGTATCGTCGTATCCCACTACCGAGATGTCCGCACCAACGCGCAGCCCGGACTCG
GTAATGGCGCGCATTGCGCCCAGCGCCATCTGATCGTTGGCAACCAGCATCGCAGTGGGAACGATGCCCTCATTCAGCAT
TTGCATGGTTTGTTGAAAACCGGACATGGCACTCCAGTCGCCTTCCCGTTCCGCTATCGGCTGAATTTGATTGCGAGTGA
GATATTTATGCCAGCCAGCCAGACGCAGACGCGCCGAGACAGAACTTAATGGGCCCGCTAACAGCGCGATTTGCTGGTGA
CCCAATGCGACCAGATGCTCCACGCCCAGTCGCGTACCGTCTTCATGGGAGAAAATAATACTGTTGATGGGTGTCTGGTC
AGAGACATCAAGAAATAACGCCGGAACATTAGTGCAGGCAGCTTCCACAGCAATGGCATCCTGGTCATCCAGCGGATAGT
TAATGATCAGCCCACTGACGCGTTGCGCGAGAAGATTGTGCACCGCCGCTTTACAGGCTTCGACGCCGCTTCGTTCTACC
ATCGACACCACCACGCTGGCACCCAGTTGATCGGCGCGAGATTTAATCGCCGCGACAATTTGCGACGGCGCGTGCAGGGC
CAGACTGGAGGTGGCAACGCCAATCAGCAACGACTGTTTGCCCGCCAGTTGTTGTGCCACGCGGTTGGGAATGTAATTCA
GCTCCGCCATCGCCGCTTCCACTTTTTCCCGCGTTTTCGCAGAAACGTGGCTGGCCTGGTTCACCACGCGGGAAACGGTC
TGATAAGAGACACCGGCATACTCTGCGACATCGTATAACGTTACTGGTTTCACATTCACCACCCTGAATTGACTCTCTTC
CGGGCGCTATCATGCCATACCGCGAAAGGTTTTGCGCCATTCGATGGTGTCCGGGATCTCGACGCTCTCCCTTATGCGAC
TCCTGCATTAGGAAGCAGCCCAGTAGTAGGTTGAGGCCGTTGAGCACCGCCGCCGCAAGGAATGGTGCATGCAAGGAGAT
GGCGCCCAACAGTCCCCCGGCCACGGGGCCTGCCACCATACCCACGCCGAAACAAGCGCTCATGAGCCCGAAGTGGCGAG
CCCGATCTTCCCCATCGGTGATGTCGGCGATATAGGCGCCAGCAACCGCACCTGTGGCGCCGGTGATGCCGGCCACGATG
CGTCCGGCGTAGAGGATCGAGATCGATCTCGATCCCGCGAAATTAATACGACTCACTATAGGGGAATTGTGAGCGGATAA
CAATTCCGTAGTCAAGGAATTAACAAAGGAGGTTTTTTATGAGCGCTTGGAGCCATCCACAATTTGAGAAGGGTGGAGGT
TCTGGCGGTGGATCGGGAGGTTCAGCGTGGAGCCACCCGCAGTTCGAAAAAGGCGCCGGATCCGTGCCTACTATCGTGAT
GGTGGACGCCTACAAGCGTTACAAGGGTAGTGGTGAAAGTGGTGAACTCACCCTCGACGGCGGCGCGGTTGCCGCGCCCG
ATCAGTACGGCGCGAAAGTCGCCGCCGAGATCCTCAAGAAGGGCGGCAACGCCGTCGACGCCGCGGTCGCCACCGCCTTC
ACCCTCGCCGTCACCTACCCCGAAGCCGGCAACATCGGCGGCGGTGGTTTCATGACCCTGTACGTCGACGGCAAGCCGTA
CTTCCTCGACTACCGCGAGATCGCGCCGAAGGCCGCGACCAAGACCATGTACCTGAACGAGAAAGGCGAGGTGATCGAGA
ACCTCAGCCTGGTCGGCGCCAAGGCCGCCGGCGTGCCGGGTACCGTGATGGGCCTATGGGAAGCGCACCAGCGCTTCGGC
AAGCTGAAGTGGAGCGAGCTGCTGACACCGGCCATCGGCTACGCGCAGACCGGCTTCAAGGTCGCCGACCAGCAGTACCA
GTACCGCCAGGACGCCATCGCGCTGTTCAACGGCAAGACCAATTTCGGCGACTACTTCGGCACCATGAAACCGGGCGAAG
TGTTCAAGCAGCCGGAACTGGCCAAGACCCTGGAGCGCATCGCCGACAAGGGCCCGGACGATTTCTACAAGGGCGAGACA
GCCAAGCTGCTCATCGCGCAGATGAAGCAGGATGGCGGCCTGATCACTTCCGACGACCTGGTCGACTACCAGGCCAAGTG
GCGCGAGCCGATGCGCATCGACTGGCAGGGCAACACCCTCTACACCGCGCCGCTGCCCAGCTCCGGCGGCATCGCCCTGG
CCCAGCTGATCGGCATCAAGGAACAGCGCGCCGCCGACTTCAAGGGCGTGGAGCTGAACTCGGCGAAGTACATCCACCTG
CTCTCGGAGATCGAGAAGCGCGTGTTCGCCGACCGCGCCGACTACCTCGGTGACCCGCAGTTCTCCAAGGTGCCGGTAGC
CCAGCTGACCGATCCGAAATACATCGCCAAGCGCGCCGGGGAAGTAAACCCGGACGCCATCTCGGCAACCGAGAAAGTCC
GCCCGGGCCTGGAGCCGCACCAGACCACGCACTTCTCCATCGTCGACAAGGACGGCAACGCCGTCAGCAACACCTACACC
CTCAACTGGGACTTCGGCAGCGGCGTGGTGGTCAAGGGCGCCGGCTTCCTGCTCAACGACGAGATGGATAACTTCAGCTC
CAAGCCGGGCGTGGCCAACGCCTTCGGCGTGGTGGGCAGCGACGCCAACGCCATCGAGCCGGGCAAGCGCATGCTCTCCT
CCATGAGCCCGAGCATCGTCACCCGCGACGGCCACGTCAGCCTGGTGCTGGGCACGCCCGGCGGTTCGCGGATCTTCACT
TCGATCTTCCAGGTGCTGAACAACGTCTACGACTTCCACCTGCCCCTGGAGAAGGCCGTGGCCGCGCAGCGTGTGCATCA
CCAACTGCTGCCGAAAGACACCATCTATTACGACGCGTACGCACCGCTCCCCGGCAAGGTCGCCGACGAGCTTAAGGCCA
TGGGCTACACCCTGGAAGATCAGGGCTGGAACATGGGTGACATCCAGGCCATCCGTGTGAACGGCAAGGCCCTGGAAACC
GCCTCCGATCCGCGCGGCCGCGGCGTCGGGATGGTGGTCAAACCCTGAGAATTCGCGCGGCCGCGGCCTAGGCGGCCTCC
TGTGTGAAATTGTTATCCGCTTTAATTAA

pDM_tsGGT$_{xe}$

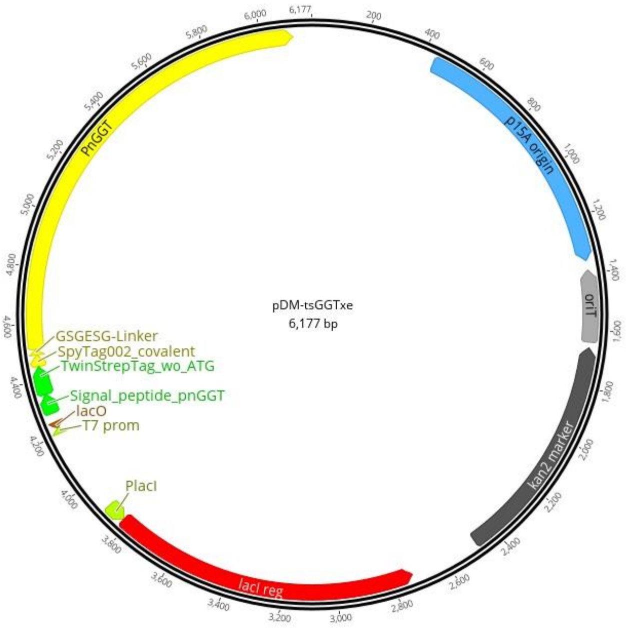

AGGCATCAAATAAAACGAAAGGCTCAGTCGAAAGACTGGGCCTTTCGTTTTATCTGTTGTTTGTCGGTGAACGCTCTCCT
GAGTAGGACAAATCCGCCGCCCTAGACAGCTGGGCGCGCCAAATACGCCCGGTAGTGATCTTATTTCATTATGGTGAAAG
TTGGAACCTCTTACGTGCCGATCAACGTCTCATTTTCGCCAAAAGTTGGCCCAGGGCTTCCCGGTATCAACAGGGACACC
AGGATTTATTTATTCTGCGAAGTGATCTTCCGTCACAGGTATTTATTCGGCGCAAAGTGCGTCGGGTGATGCTGCCAACT
TACTGATTTAGTGTATGATGGTGTTTTTGAGGTGCTCCAGTGGCTTCTGTTTCTATCAGCTGTCCCTCCTGTTCAGCTAC
TGACGGGGTGGTGCGTAACGGCAAAAGCACCGCCGGACATCAGCGCTAGCGGAGTGTATACTGGCTTACTATGTTGGCAC
TGATGAGGGTGTCAGTGAAGTGCTTCATGTGGCAGGAGAAAAAAGGCTGCACCGGTGCGTCAGCAGAATATGTGATACAG
GATATATTCCGCTTCCTCGCTCACTGACTCGCTACGCTCGGTCGTTCGACTGCGGCGAGCGGAAATGGCTTACGAACGGG
GCGGAGATTTCCTGGAAGATGCCAGGAAGATACTTAACAGGGAAGTGAGAGGGCCGCGGCAAAGCCGTTTTTCCATAGGC
TCCGCCCCCCTGACAAGCATCACGAAATCTGACGCTCAAATCAGTGGTGGCGAAACCCGACAGGACTATAAAGATACCAG
GCGTTTCCCCCTGGCGGCTCCCTCGTGCGCTCTCCTGTTCCTGCCTTTCGGTTTACCGGTGTCATTCCGCTGTTATGGCC
GCGTTTGTCTCATTCCACGCCTGACACTCAGTTCCGGGTAGGCAGTTCGCTCCAAGCTGGACTGTATGCACGAACCCCCC
GTTCAGTCCGACCGCTGCGCCTTATCCGGTAACTATCGTCTTGAGTCCAACCCGGAAAGACATGCAAAAGCACCACTGGC
AGCAGCCACTGGTAATTGATTTAGAGGAGTTAGTCTTGAAGTCATGCGCCGGTTAAGGCTAAACTGAAAGGACAAGTTTT
GGTGACTGCGCTCCTCCAAGCCAGTTACCTCGGTTCAAAGAGTTGGTAGCTCAGAGAACCTTCGAAAAACCGCCCTGCAA
GGCGGTTTTTTCGTTTTCAGAGCAAGAGATTACGCGCAGACCAAAACGATCTCAAGAAGATCATCTTATTAATCAGATAA
AATATTTCTAGCATGAGCGGATACATATTTGAATGTATTTAGAAAAATAAACAAATAGGGGTTCCGCGCACATTTCCCCG
AAAAGTGCCACCTGCTAGGGCCGGCCTACGGCCAGCCTCGCAGAGCAGGATTCCCGTTGAGCACCGCCAGGTGCGAATAA
GGGACAGTGAAGAAGGAACACCCGCTCGCGGGTGGGCCTACTTCACCTATCCTGCCCGGCTGACGCCGTTGGATACACCA
AGGAAAGTCTACACGAACCCTTTGGCAAAATCCTGTATATCGTGCGAAAAAGGATGGATATACCGAAAAAATCGCTATAA
TGACCCCGAAGCAGGGTTATGCAGCGGAAAAGGACAACGCGCGGACCGCGGTCCAATTAATTATTAGAAAAATTCATCCA
GCATCAGATGAAATTGCAGTTTGTTCATATCCGGATTATCAATGCCATATTTCTGAAACAGACGTTTTTGCAGGCTCGGG
CTAAATTCGCCCAGGCAGTTCCACAGAATGGCCAGATCCTGATAACGATCCGCAATGCCCACACGGCCCACATCAATGCA
GCCAATCAGTTTGCCTTCATCGAAAATCAGGTTATCCAGGCTAAAATCGCCGTGGGTCACCACGCTATCCGGGCTAAACG
GCAGCAGTTTATGCATTTCTTTCCACACCTGTTCCACCGGCCAGCCGTTACGTTCATCATCAAAATCGCTCGCATCCACC
AGGCCGTTGTTCATACGGCTCTGCGCCTGGGCCAGACGAAACACACGATCGCTGTTAAACGGGCAGTTGCACACCGGAAT
GCTATGCAGACGACGCAGAAACACGGCCAGCGCATCCACAATGTTTTCGCCGCTATCCGGATATTCTTCCAGCACCTGAA
ACGCGGTTTTGCCCGGAATCGCGGTGGTCAGCAGCCACGCATCATCCGGGGTGCGAATAAAATGTTTAATGGTCGGCAGC
GGCATAAATTCGGTCAGCCAGTTCAGACGCACCATTTCATCGGTCACATCGTTCGCCACGCTGCCTTTGCCATGTTTCAG
AAACAGTTCCGGCGCATCCGGTTTGCCATACAGACGATAAATGGTCGCGCCGCTCTGACCCACGTTATCACGCGCCCATT
TATAGCCATACAGATCCGCATCCATGTTGCTGTTCAGACGCGGACGGCTACAGCTCGTTTCACGCTGAATATGGCTCATA
ACACCCCTTGTATTACTGTTTATGTAAGCAGACAGTTTTATTGTTCATGATGATATATTTTTATCTTGTGCAATGTAACA
TCAGAGATTTTGAGACACAAATTTAAATCGTAATTATTGGGGACCCCTGGATTCTCACCAATAAAAAACGCCCGGCGGCA
ACCGAGCGTTCTGAACAAATCCAGATGGAGTTCTGAGGTCATTACTGGATCTATCAACAGGAGTCCAAGACTAGTTTAAT

TGCGTTGCGCTCACTGCCCGCTTTCCAGTCGGGAAACCTGTCGTGCCAGCTGCATTAATGAATCGGCCAACGCGCGGGGA
GAGGCGGTTTGCGTATTGGGCGCCAGGGTGGTTTTTCTTTTCACCAGTGAGACGGGCAACAGCTGATTGCCCTTCACCGC
CTGGCCCTGAGAGAGTTGCAGCAAGCGGTCCACGCTGGTTTGCCCCAGCAGGCGAAAATCCTGTTTGATGGTGGTTAACG
GCGGGATATAACATGAGCTGTCTTCGGTATCGTCGTATCCCACTACCGAGATGTCCGCACCAACGCGCAGCCCGGACTCG
GTAATGGCGCGCATTGCGCCCAGCGCCATCTGATCGTTGGCAACCAGCATCGCAGTGGGAACGATGCCCTCATTCAGCAT
TTGCATGGTTTGTTGAAAACCGGACATGGCACTCCAGTCGCCTTCCCGTTCCGCTATCGGCTGAATTTGATTGCGAGTGA
GATATTTATGCCAGCCAGCCAGACGCAGACGCGCCGAGACAGAACTTAATGGGCCCGCTAACAGCGCGATTTGCTGGTGA
CCCAATGCGACCAGATGCTCCACGCCCAGTCGCGTACCGTCTTCATGGGAGAAAATAATACTGTTGATGGGTGTCTGGTC
AGAGACATCAAGAAATAACGCCGGAACATTAGTGCAGGCAGCTTCCACAGCAATGGCATCCTGGTCATCCAGCGGATAGT
TAATGATCAGCCCACTGACGCGTTGCGCGAGAAGATTGTGCACCGCCGCTTTACAGGCTTCGACGCCGCTTCGTTCTACC
ATCGACACCACCACGCTGGCACCCAGTTGATCGGCGCGAGATTTAATCGCCGCGACAATTTGCGACGGCGCGTGCAGGGC
CAGACTGGAGGTGGCAACGCCAATCAGCAACGACTGTTTGCCCGCCAGTTGTTGTGCCACGCGGTTGGGAATGTAATTCA
GCTCCGCCATCGCCGCTTCCACTTTTTCCCGCGTTTTCGCAGAAACGTGGCTGGCCTGGTTCACCACGCGGGAAACGGTC
TGATAAGAGACACCGGCATACTCTGCGACATCGTATAACGTTACTGGTTTCACATTCACCACCCTGAATTGACTCTCTTC
CGGGCGCTATCATGCCATACCGCGAAAGGTTTTGCGCCATTCGATGGTGTCCGGGATCTCGACGCTCTCCCTTATGCGAC
TCCTGCATTAGGAAGCAGCCCAGTAGTAGGTTGAGGCCGTTGAGCACCGCCGCCGCAAGGAATGGTGCATGCAAGGAGAT
GGCGCCCAACAGTCCCCCGGCCACGGGGCCTGCCACCATACCCACGCCGAAACAAGCGCTCATGAGCCCGAAGTGGCGAG
CCCGATCTTCCCCATCGGTGATGTCGGCGATATAGGCGCCAGCAACCGCACCTGTGGCGCCGGTGATGCCGGCCACGATG
CGTCCGGCGTAGAGGATCGAGATCGATCTCGATCCCGCGAAATTAATACGACTCACTATAGGGGAATTGTGAGCGGATAA
CAATTCCTAGTCTCGTACGACATTTAAGGAGGTTTATATGCGCGTTTTCCACTTCAGCAAACTTCCCTTGGGTGTGGCAA
TCCTGGCCGCAAGTTCGTCAGTGTTTGCGAGCGCTTGGAGCCATCCACAATTTGAGAAGGGTGGAGGTTCTGGCGGTGGA
TCGGGAGGTTCAGCGTGGAGCCACCCGCAGTTCGAAAAAGGCGCCGGATCCGTGCCTACTATCGTGATGGTGGACGCCTA
CAAGCGTTACAAGGGTAGTGGTGAAAGTGGTGAACTCACCCTCGACGGCGGCGCGGTTGCCGCGCCCGATCAGTACGGCG
CGAAAGTCGCCGCCGAGATCCTCAAGAAGGGCGGCAACGCCGTCGACGCCGCGGTCGCCACCGCCTTCACCCTCGCCGTC
ACCTACCCCGAAGCCGGCAACATCGGCGGCGGTGGTTTCATGACCCTGTACGTCGACGGCAAGCCGTACTTCCTCGACTA
CCGCGAGATCGCGCCGAAGGCCGCGACCAAGACCATGTACCTGAACGAGAAAGGCGAGGTGATCGAGAACCTCAGCCTGG
TCGGCGCCAAGGCCGCCGGCGTGCCGGGTACCGTGATGGGCCTATGGGAAGCGCACCAGCGCTTCGGCAAGCTGAAGTGG
AGCGAGCTGCTGACACCGGCCATCGGCTACGCGCAGACCGGCTTCAAGGTCGCCGACCAGCAGTACCAGTACCGCCAGGA
CGCCATCGCGCTGTTCAACGGCAAGACCAATTTCGGCGACTACTTCGGCACCATGAAACCGGGCGAAGTGTTCAAGCAGC
CGGAACTGGCCAAGACCCTGGAGCGCATCGCCGACAAGGGCCCGGACGATTTCTACAAGGGCGAGACAGCCAAGCTGCTC
ATCGCGCAGATGAAGCAGGATGGCGGCCTGATCACTTCCGACGACCTGGTCGACTACCAGGCCAAGTGGCGCGAGCCGAT
GCGCATCGACTGGCAGGGCAACACCCTCTACACCGCGCCGCTGCCCAGCTCCGGCGGCATCGCCCTGGCCCAGCTGATCG
GCATCAAGGAACAGCGCGCCGCCGACTTCAAGGGCGTGGAGCTGAACTCGGCGAAGTACATCCACCTGCTCTCGGAGATC
GAGAAGCGCGTGTTCGCCGACCGCGCCGACTACCTCGGTGACCCGCAGTTCTCCAAGGTGCCGGTAGCCCAGCTGACCGA
TCCGAAATACATCGCCAAGCGCGCCGGGGAAGTAAACCCGGACGCCATCTCGGCAACCGAGAAAGTCCGCCCGGGCCTGG
AGCCGCACCAGACCACGCACTTCTCCATCGTCGACAAGGACGGCAACGCCGTCAGCAACACCTACACCCTCAACTGGGAC
TTCGGCAGCGGCGTGGTGGTCAAGGGCGCCGGCTTCCTGCTCAACGACGAGATGGATAACTTCAGCTCCAAGCCGGGCGT
GGCCAACGCCTTCGGCGTGGTGGGCAGCGACGCCAACGCCATCGAGCCGGGCAAGCGCATGCTCTCCTCCATGAGCCCGA
GCATCGTCACCCGCGACGGCCACGTCAGCCTGGTGCTGGGCACGCCCGGCGGTTCGCGGATCTTCACTTCGATCTTCCAG
GTGCTGAACAACGTCTACGACTTCCACCTGCCCCTGGAGAAGGCCGTGGCCGCGCAGCGTGTGCATCACCAACTGCTGCC
GAAAGACACCATCTATTACGACGCGTACGCACCGCTCCCCGGCAAGGTCGCCGACGAGCTTAAGGCCATGGGCTACACCC
TGGAAGATCAGGGCTGGAACATGGGTGACATCCAGGCCATCCGTGTGAACGGCAAGGCCCTGGAAACCGCCTCCGATCCG
CGCGGCCGCGGCGTCGGGATGGTGGTCAAACCCTGAGAATTCGCGCGGCCGCGGCCTAGGCGGCCTCCTGTGTGAAATTG
TTATCCGCTTTAATTAA

pDM_tsGGT$_{xe}$_D386N: pDM_tsGGT$_{xe}$ aa position 386 GAC → AAC

pDM_tsGGT$_{xe}$_T364A: pDM_tsGGT$_{xe}$ aa position 364 ACC → GCG

Supplementary Code

Python Code dedicated to automatically process MS/MS data derived from a QTRAP 4000 mass spectrometer (Sciex) as described in Material and Methods. The obtained raw data file ".wiff" (Sciex) was converted with msConvert[42] (ProteoWizard, Version: 3.0.19217-f7f3a630b) into rich ".txt" files. The ".txt" files serve as input for the script, which generates a .csv spreadsheet, containing sample name, measuring time point in sec relative to start time and integrated peak area of analyte and internal standard.

```
import os
import re
import peakutils
import numpy as np
import matplotlib.pyplot as plt



class MSAnalyzer:

    def __init__(self) -> None:
        self.store = {}

    def __call__(self) -> None:
        """Start processing txt files"""
        self.create_folders(['data', 'csv'])
        file_names = os.listdir('./data')
        for file_name in file_names:
            if file_name.endswith('.txt'):
                key = self.correcting_file_name(file_name)
                with open(f'./data/{file_name}', 'r') as f:
                    raw_data = f.readlines()
                    self.get_time(raw_data, key)
                    self.get_ions(raw_data, key)
                    self.get_startpoint(raw_data, key)
                    self.calc_area_under_curve(key)
                    self.plot_integrated_peak(key)
                    self.export_to_csv(key)
```

```
    @staticmethod
    def create_folders(folders: list) -> None:
        """Check and create folders"""
        for folder in folders:
            os.makedirs(folder, exist_ok=True)
        if len(os.listdir('./data')) == 0:
            raise FileNotFoundError('Expected ".txt" files in data
folder')


    @staticmethod
    def convert_to_seconds(time_str: str) -> int:
        """Convert time to seconds"""
        h, m, s = time_str.split(':')
        return int(h) * 3600 + int(m) * 60 + int(s)


    @staticmethod
    def correcting_file_name(file_name: str) -> str:
        """Clean up file name numbering"""
        file_name, _ = os.path.splitext(file_name)
        findings = re.findall(r'\(\d+\)', file_name)

        # get number between ()
        if not findings:
            return f'{file_name.strip()}0'
        if len(findings) == 1:
            tmp = findings[0]
            number = tmp[1:-1]  # strip away ()
            file_name = file_name.replace(findings[0], '').strip()
```

```
            return f'{file_name}{number}'
        raise ValueError(f'Not able to handle file number findings:
{findings}')



    def get_time(self, raw_data: list, key: str) -> None:
        """Read time from data"""
        for line in raw_data:
            if 'id: TIC' in line:
                y = raw_data[raw_data.index(line) + 5]
        y_clean = y[24:len(y) - 2].split(' ')
        tmp = []
        for i in y_clean:
            tmp.append(float(i))
        self.store[f'{key}_time'] = np.array(tmp)



    def get_startpoint(self, raw_data: list, key: str) -> None:
        """Get the start time point for enzyme kinetics"""
        for line in raw_data:
            if 'startTimeStamp:' in line:
                measuring_date = raw_data[raw_data.index(line)]
        measuring_time = measuring_date[31:len(measuring_date) - 2]
        self.store[f'{key}_timepoint'] =
self.convert_to_seconds(measuring_time)



    def get_ions(self, raw_data: list, key: str) -> None:
        """Read ion count"""
        srms = []
        quadro = []
        for line in raw_data:
```

```
            self.counter = 0
            if 'id: TIC' in line:
                y = raw_data[raw_data.index(line) + 8]
                yclean = y[24:len(y) - 2].split(' ')
                tmp = []
                for i in yclean:
                    tmp.append(float(i))
                self.store[f'{key}_ion_total'] = np.array(tmp)

            if 'id: - SRM SIC' in line:
                srms.append(raw_data[raw_data.index(line) + 20])
                quadro.append(line)

        self.store[f'{key}_srm_names'] = []
        for measure in quadro:
            z = measure[0:len(measure)-1]
            self.store[f'{key}_srm_names'].append(z)

        self.store[f'{key}_srms'] = []
        for srm in srms:
            y = srm[24:len(srm) - 2].split(' ')
            tmp = []
            for i in y:
                tmp.append(float(i))
            self.store[f'{key}_srms'].append(np.array(tmp))




    def calc_area_under_curve(self, key: str) -> None:
        """Calculate area under curve"""
        #time = self.store[f'{key}_time']
        self.store[f'{key}_int_start'] = 0.0  # defines at what
point the peak integration starts, depends on the dead volume of the
setup
        self.store[f'{key}_int_end'] = 0.2  #  defines at what point
the peak integration ends (typically right after the peak)
```

```
        self.store[f'{key}_srm'] = []
        self.store[f'{key}_counter'] = 0
        self.store[f'{key}_signal_corrected'] = []
        self.store[f'{key}_base'] = []
        self.store[f'{key}_normalized'] = []
        self.store[f'{key}_int_time'] = []

        for srm in self.store[f'{key}_srms']:
            self.store[f'{key}_base'].append(peakutils.baseline(srm,
max_it=20, deg=3, tol=0.1))  # degree of polynomial fit
            self.store[f'{key}_signal_corrected'].append(srm -
self.store[f'{key}_base'][self.store[f'{key}_counter']])  #
substract fitted baseline from signal
            y_cut =
self.store[f'{key}_signal_corrected'][self.store[f'{key}_counter']][
np.logical_and(self.store[f'{key}_time'] >=
self.store[f'{key}_int_start'],self.store[f'{key}_time'] <=
self.store[f'{key}_int_end'])]  # chop the baseline adjusted graph
by using int_start, int_end boundaries
            self.store[f'{key}_srm'].append(y_cut)

self.store[f'{key}_int_time'].append(self.store[f'{key}_time'][np.lo
gical_and(self.store[f'{key}_time'] >=
self.store[f'{key}_int_start'],self.store[f'{key}_time'] <=
self.store[f'{key}_int_end'])]) # adjust the time array to be within
the integration barriers int_start and int_end
            srm_area = int(np.sum(y_cut))  # Integrate the baseline
adjusted area between int_start and int_end
            self.store[f'{key}_normalized'].append(srm_area)
            self.store[f'{key}_counter'] += 1




    def plot_integrated_peak(self, key: str) -> None:
        for i in range(self.store[f'{key}_counter']):
            plt.title({self.store[f'{key}_srm_names'][i]})
```

```
            plt.plot(self.store[f'{key}_time'],
self.store[f'{key}_srms'][i])

plt.plot(self.store[f'{key}_int_time'][i],self.store[f'{key}_srm'][i
])
            plt.plot(self.store[f'{key}_time'],
self.store[f'{key}_base'][i])
            plt.show()




    def export_to_csv(self, key: str) -> None:
        with open(os.path.join('csv', 'results.csv'), 'a') as
csv_out:

csv_out.write('sample_name;time(s);int_start;int_end;srm_name;area\n
')
            for i in range(self.store[f'{key}_counter']):

csv_out.write(f"{key};{self.store[f'{key}_timepoint']};{self.store[f
'{key}_int_start']};{self.store[f'{key}_int_end']};{(self.store[f'{k
ey}_srm_names'][i])};{self.store[f'{key}_normalized'][i]}\n")


if __name__ == '__main__':
    analyzer = MSAnalyzer()
    analyzer()
```
